# Theory and evidence of amplitude control by frequency detuning in a coupled neuronal oscillator system

**DOI:** 10.1371/journal.pcbi.1014686

**Published:** 2026-08-25

**Authors:** Adam C. Lu, Seyed AmirHossein Ourang, Jeffrey D. Moore

**Affiliations:** 1 Department of Biological Sciences, Section of Neurobiology, Dornsife College of Letters, Arts and Sciences, University of Southern California, Los Angeles, United States of America; 2 Children’s Hospital Los Angeles, Los Angeles, United States of America; 3 Craniofacial Biology PhD Program, Ostrow School of Dentistry, University of Southern California, Los Angeles, United States of America; The University of Edinburgh, UNITED KINGDOM OF GREAT BRITAIN AND NORTHERN IRELAND

## Abstract

Neuronal oscillator circuits that generate rhythmic movements must operate flexibly and reliably to produce the varied motor patterns that animals exhibit naturally. Rodents rhythmically “whisk” their vibrissae for haptic perception, and they dynamically adjust the whisking range to serve different perceptual goals. Whisking is controlled by a brainstem oscillator circuit that is coupled to breathing, yet how whisking amplitude is modulated remains unknown. Here we propose and evaluate an amplitude control mechanism based on principles of synchronization in coupled oscillators. Specifically, a re-analysis of rat behavioral data demonstrates that whisking exhibits kinematic signatures and phase dynamics of amplification via entrainment with “sniffing”, a mode of high-frequency breathing. A neuronal network model of the whisking oscillator circuit suggests that whisking amplitude can be modulated by shifting the oscillator’s intrinsic frequency relative to the sniffing frequency, analogous to the engineering technique of “detuning”. Based on these results, we propose that detuning between coupled neuronal oscillators may represent a general computational strategy for gain control in nervous systems.

## Author summary

Animals generate a multitude of rhythmic movement behaviors that enable them to interact with their environment, such as ventilating, locomoting, eating, and active sensing. Though these movements are highly structured, animals must implement them flexibly and adaptively to meet the demands of the environment. How do networks of neurons that generate the oscillatory neuronal activity for these movements enable this important flexibility? Here we focus on rodents’ use of their vibrissae (whiskers) to explore their environment by rhythmically scanning the tactile landscape around their faces. Through quantitative analyses of behavioral data and computational modeling, we show that rodents can exploit the difference in frequency between two interacting neuronal oscillator networks to dynamically adjust the size of their vibrissae movements, much like tuning a radio toward or away from a station adjusts the signal strength. Beyond the neuronal control of movement, interactions between brain rhythms with different underlying frequencies seem to be a ubiquitous phenomenon observed in studies of diverse cognitive processes. Based on such observations, we suggest that frequency-based amplitude control like this may represent a more general computational strategy utilized by nervous systems.

## Introduction

Animal movement requires the nervous system to produce temporally structured activations of multiple muscle groups to generate coordinated, goal-directed motor commands. The most primordial types of movements, including ventilation, digestion, and locomotion, are oscillatory. Such basic movement sequences are thought to be produced by central pattern generators (CPGs), defined as networks of neurons whose activity can generate oscillatory movements with correct timing and sequences in the absence of sensory feedback [1]. Though these types of movement are considered highly structured and automatic, their kinematics can vary substantially to meet an animal’s objectives. For example, an animal can locomote with different gaits or swimming patterns [2] and breathe with different rates [3] and tidal volumes. How do the neuronal architectures of CPGs enable them to operate with the adaptability to generate the variety of motor patterns that animals exhibit in nature, while still respecting the biomechanical constraints of the motor plant?

**Fig 1:**
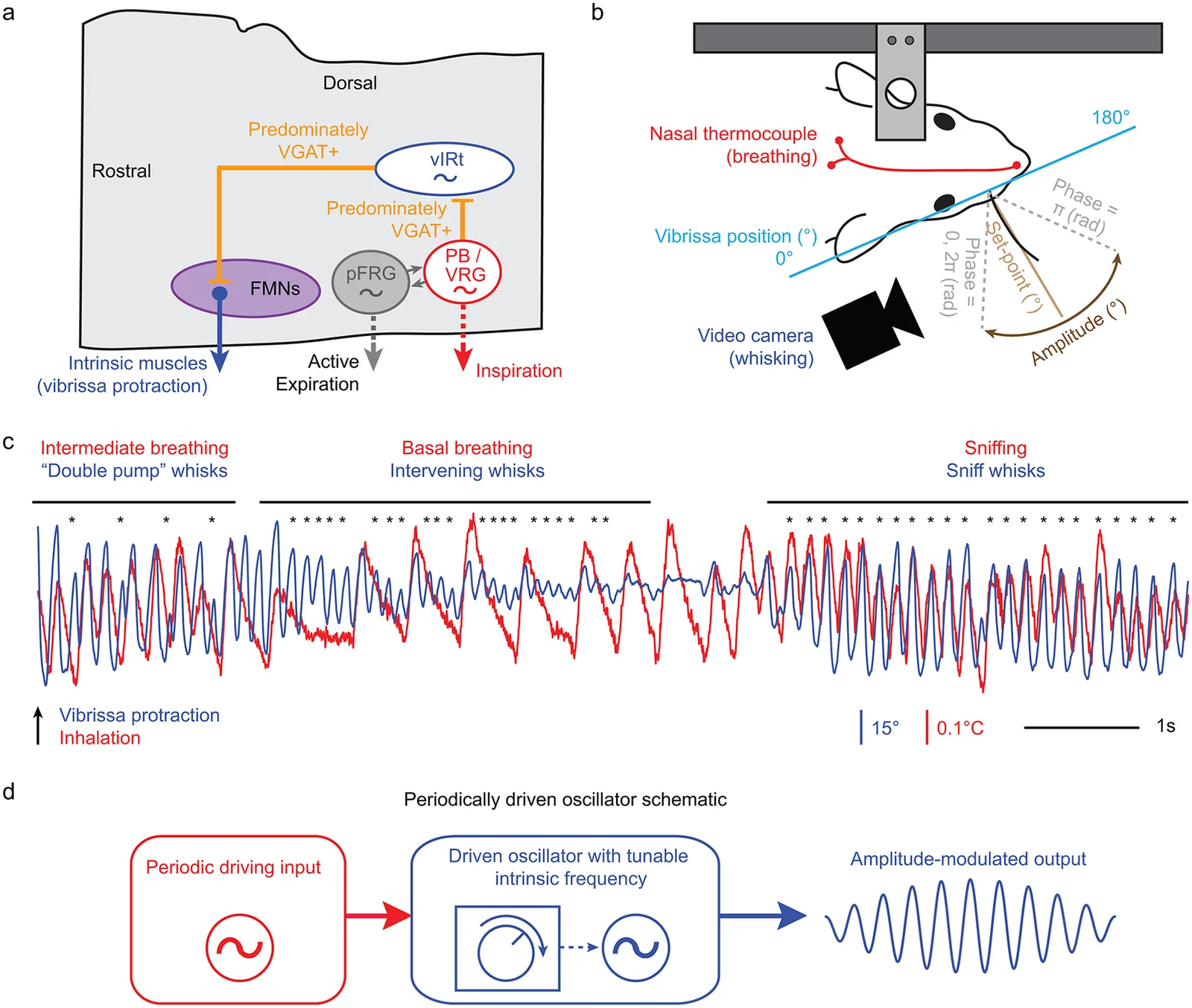
Whisking and breathing CPG circuit comparison to a driven oscillator system. **(a)** Schematic of the whisking CPG circuit and its inputs from the ventral respiratory column. Connectivity is defined in [14,15,25]. Abbreviations: vIRt: vibrissa intermediate reticular formation; PB: preBӧtzinger complex; VRG: ventral respiratory group; pFRG: parafacial respiratory group; FMNs: facial motor neurons; *VGAT*: vesicular inhibitory amino acid transporter. **(b)** Experimental setup that was used to simultaneously monitor vibrissa movements with a camera (blue) and breathing via a thermocouple implanted in the nasal cavity (red) in rats. Described in [14]. Whisking kinematic conventions and definitions of vibrissa position (angle, light blue), whisking amplitude (angular range, brown), whisking set-point (angular mid-point, tan), and phase in the whisk cycle (gray) are shown. **(c)** Example traces of vibrissa movement (blue) and intranasal temperature changes (red). Upward deflections correspond to vibrissa protraction and temperature decreases that indicate inhalation. Previously described modes of whisking are annotated (*). **(d)** Schematic of interactions between oscillators in a periodically driven oscillator system in which the driven oscillator has a tunable intrinsic frequency.

To address this important question, we focus on a CPG for oscillatory orofacial movement in rodents as a model system. As with ventilation, digestion, and locomotion, mammalian orofacial behaviors, including chewing, licking, lapping, breathing, and suckling, are also oscillatory and controlled by CPGs [4]. CPGs for some of these behaviors, including breathing, licking, and lapping, have been shown to involve neuronal networks in the reticular formation of the medulla [4,5]. Additionally, rodent species have large tactile hairs on the snout, called vibrissae, that they sweep back and forth to scan the physical environment around the face to detect, locate, and palpate objects and surfaces for haptic perception [6,7]. This sweeping motion, termed “whisking” [8], together with rapid breathing and naris movement for olfactory sampling known as “sniffing”, comprise a prominent mode of exploratory behavior for rodents [3,9,10]. Consistent with control by CPGs, these movements occur in the absence of sensory feedback [3,11]. Sniffing and whisking are biomechanically linked, as the snout muscles for naris movements also move the vibrissae [12]. Correspondingly, whisking and breathing are highly synchronized across the full range of breathing frequencies, and a CPG for whisking has been identified in the vibrissa intermediate reticular formation (vIRt) of the medulla, a region that receives input from a nearby neuronal oscillator circuit for breathing [13–15] (Fig 1a). This circuitry likely underlies the observed relative coordination [2] between whisking and breathing to conform to biomechanical constraints. Although the sequence of muscle activations in whisking is highly stereotyped [16], the whisking kinematics are dynamic; that is, rodents vary the frequency, amplitude, and set-point of their vibrissa movements (Fig 1b) in different behavioral contexts [17,18]. In rats, large-amplitude whisking in the range of 5–11 Hz accompanies exploration while small-amplitude and protracted whisking with spectral power up to 25 Hz is associated with palpating objects for discrimination [3,19,20]. How do interactions between whisking and breathing oscillators support this wide dynamic range of vibrissa movements?

Here we address the specific question of how the rodent nervous system can dynamically modulate whisking amplitude. Curiously, although sniffing and whisking are tightly phase-locked, rats and mice exhibit prominent “intervening whisks” between breaths during slow breathing [14], which would seem to be counterproductive to maintaining synchrony for biomechanical [12] or perceptual [21] simplicity. We hypothesize that the amplitudes of these intervening whisks are indicative of a coupled oscillator system in which effects of perturbations to the whisking oscillator from the breathing CPG persist over multiple whisks, qualitatively analogous to underdamped, driven linear oscillator systems. In driven oscillator systems in general, persistence of perturbation effects across multiple cycles enables frequency-selective amplification when the drive frequency is aligned with the driven oscillator’s “intrinsic” frequency, that is, the dominant oscillation frequency in the absence of external periodic drive. This frequency selectivity arises through constructive interference, where effects of past drive cycles reinforce effects of subsequent drive cycles near the intrinsic frequency. In linear systems such as the well-characterized driven harmonic oscillator, this constructive interference amplification is termed “resonance” and arises due to the accumulation of stored energy. In driven nonlinear oscillators such as networks of neurons, phase alignment to the drive signal, known as “entrainment”, leads to a qualitatively similar frequency-dependent amplification [22]. This phase alignment can occur when the drive and intrinsic frequencies occur at near rational ratios, corresponding to distinct N:M integer frequency locking modes, which are observable when the drive strength is within an appropriate range [22,23]. We therefore postulated that the closely matched dominant frequencies of the neuronal oscillators controlling whisking and sniffing enable them to operate within the 1:1 locking mode, where whisking amplitude is maximal when the intrinsic whisking oscillator frequency matches the sniffing frequency. A corollary to this postulate is that if the animal is able to dynamically modulate the intrinsic frequency of either or both oscillators, then it can control whisking amplitude by adjusting the frequency difference between the oscillator networks, an algorithm analogous to “detuning” in engineering systems [22,23]. This hypothesis predicts that whisking should have kinematic signatures and phase dynamics reflective of entrainment to sniffing.

We re-analyzed a publicly available database of whisking and breathing signals in head-restrained rats [14,24] (Fig 1b, 1c) and identified kinematic features consistent with this hypothesis; however, because breathing is always occurring, the intrinsic dynamics of the vIRt network in isolation are not easily observable in experimental animal models. Therefore, to assess the plausibility of the “detuning” hypothesis, we constructed a biologically constrained neuronal network model of the whisking CPG in the vIRt, based on prior experimental observations and modeling studies [14,15,24,25], and we simulated how this model responds to breathing control signals at varying frequencies. We show that the simulated whisking amplitude is maximized when the intrinsic vIRt frequency matches the breathing frequency, and that the resulting whisking signals have key kinematic signatures and phase dynamics that mimic the behavioral observations in rats [14]. These findings demonstrate that frequency detuning is a biologically plausible algorithm for achieving amplitude modulation in a coupled neuronal oscillator system for controlling physical movement. Intriguingly, the use of entrainment and detuning for neural computation has recently been proposed as a mechanism involved in cognitive processing [23], suggesting that this model may represent a more general computational strategy that could be implemented throughout the nervous system.

## Detailed background

Since the initial discovery of a whisking CPG in the vIRt [14], there has been rapid scientific progress in delineating the structure and function of rhythmic whisking control in a series of studies over the last decade [15,24,25]. First, whisking in rats can occur during transient periods of apnea, suggesting that whisking and breathing are controlled by separable neuronal oscillators. Second, whisking can occur during basal (slow) breathing as well as high-frequency sniffing. During basal breathing, whisking and breathing occur at incommensurate frequencies, and the phase of whisking is reset when an inhalation occurs. In contrast, whisking does not appear to influence the breathing rhythm, suggesting a unidirectional coupling between the whisking and breathing CPGs [14,25] (Fig 1a). Spiking rates of neurons in the vIRt are phase-locked with whisking, even during periods of basal breathing, such that approximately 2/3 of neurons that were measured fire in phase with retraction (${\text{vIRt}}^{\text{ret}}$), and ~1/3 fire in phase with protraction (${\text{vIRt}}^{\text{pro}}$) [14,15,25]. Injections of the glutamatergic agonist kainic acid near the vIRt produce sustained ipsilateral whisking in the lightly anesthetized rat [14] or mouse [26] concomitant with rhythmic spiking in the vIRt [25]. Lesioning the vIRt electrolytically or blocking the release of neurotransmitter from vIRt parvalbumin-positive (PV+) neurons prevents ipsilateral whisking and results in the ipsilateral vibrissae being maintained in a tonically protracted state [14,15]. The vIRt receives predominantly inhibitory input from the central rhythm generator for inhalation in the preBӧtzinger complex (PB) [15], and sends predominantly inhibitory outputs to the vibrissa motor neurons in the facial nucleus [14,15] (Fig 1a). Though whisking has previously been characterized by alternating activations of intrinsic vibrissa protractor muscles and extrinsic mystacial pad retractor muscles [16,19,27], the lack of extrinsic muscle activity during intervening whisks suggests that the vIRt likely targets only the facial motor neurons (FMNs) for the intrinsic muscles [14]. The observed 1:1 coordination between active protraction and retraction is likely due to direct activation of retractor muscles by the breathing CPG during sniffing, likely through the parafacial respiratory group [14,25] (Fig 1a). Together, these results demonstrate that networks of neurons in the vIRt are necessary and sufficient to generate patterned whisking, and they suggest that the inputs from PB could enable the observed phase resetting during inhalation.

Specifically, inhibitory input from PB during inhalation could directly innervate inhibitory ${\text{vIRt}}^{\text{ret}}$ neurons resulting in immediate disinhibition of FMNs that result in protraction. A parsimonious computational model of the whisking CPG circuit in the vIRt proposes that a rhythmic oscillation intrinsic to the vIRt arises from inhibitory interactions between ${\text{vIRt}}^{\text{pro}}$ and ${\text{vIRt}}^{\text{ret}}$ neuron populations, which also have adaptation currents [24], an extension of the half-center oscillator model for motor pattern generation [28,29]. This vIRt model accounts for the observed intervening whisks (Fig 1a, 1c), the phase response of the whisking rhythm to low-frequency inhalations [14], and the observation that the first whisk following inhalation is larger than subsequent intervening whisks. This model also explains the special case of “double pumps” observed in the whisking rhythm [30], which occur at intermediate breathing frequencies where there is one driven and one intervening whisk per breath (Fig 1c). Experimental and theoretical evidence suggests that the observed 1:1 coordination between whisks and sniffs results from the vIRt network being reset on every sniff cycle, thus entraining the intrinsic vIRt rhythm to sniffing [14,22,24] (Fig 1c), providing evidence for what von Holst described as the “magnet effect” in observations of fin patterns in fishes as early as the 1930s, where one motor rhythm subsumes another [2].

Intriguingly, these observations of the various patterns of whisking and breathing movements (Fig 1c) and the observed anatomical connections in the ventral medulla (Fig 1a) are consistent with a driven oscillator system (Fig 1d) in which perturbations to the whisking oscillator in vIRt by breathing drive from PB persist across multiple cycles. This driven oscillator structure implies that oscillation amplitude can be modulated by changing the drive frequency and/or the intrinsic frequency of the driven oscillator for amplitude control. Specifically, here we propose that the rodent whisking CPG implements frequency-dependent amplification via entrainment to sniffing and modulation of the vIRt intrinsic frequency within the 1:1 frequency locking mode. Further, we propose that descending control of the vIRt intrinsic frequency provides a tunable parameter for amplitude modulation (Fig 1d). The convergent results from quantitative analysis of whisking/breathing dynamics and from computational modeling described below are consistent with the hypothesis that the vibrissa motor system has implemented such a detuning-based amplitude control strategy.

## Results

### Kinematic features of whisking in relation to breathing

**Fig 2:**
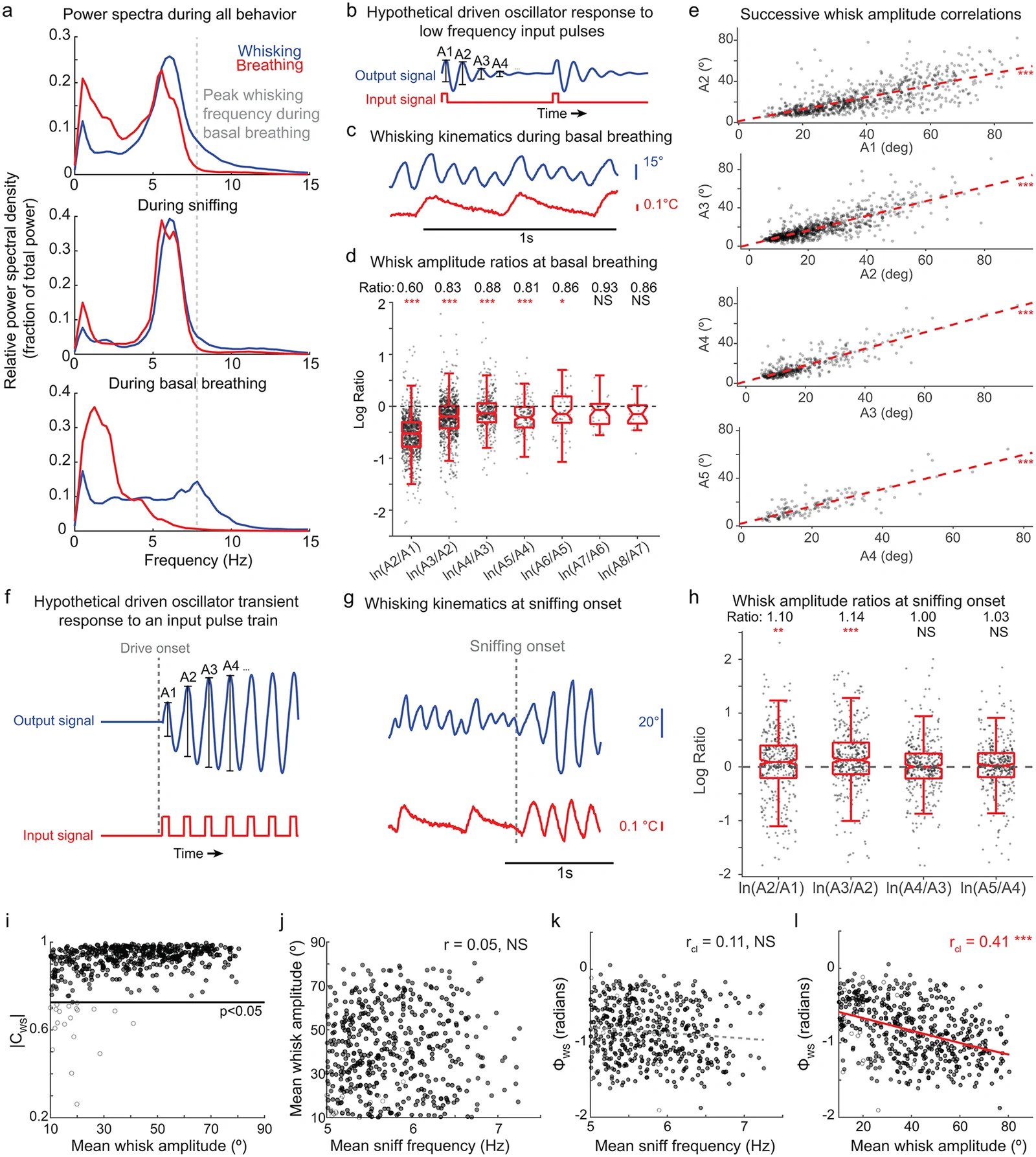
Kinematic features of rhythmic whisking are consistent with entrainment to breathing. (a) Power spectra of whisking and breathing signals as shown in Fig 1c over all behavioral epochs (top), during periods of sniffing only (middle), and during basal breathing only (bottom). Epoch-averaged power spectra are computed on 2-second non-overlapping segments with a time-bandwidth product of 1 and 1 taper (b) Hypothetical input (red) and output (blue) signals of a periodically driven oscillator system that exhibits decaying amplitude oscillations in response to driving input pulses at a frequency below the driven oscillator’s intrinsic frequency. This schematic illustrates how amplitudes of successive oscillation cycles are calculated (see **Materials and Methods**) (c) Example intervening whisks during basal respiration, blowup of a portion of the trace in Fig 1c. (d) Distributions of log ratios $d_{n+\text{1},n}=\;\text{ln}\left(A_{n+\text{1}}/A_{n}\right)\;$of successive whisk amplitudes, where A represents the amplitude and n represents the nth whisk after an inhalation, for all whisks during basal breathing, as defined in the schematic in panel b. Red lines inside boxes indicate the median log ratios $\tilde{d_{n+\text{1},n}}$ over all breaths, red notches indicate 95% confidence intervals of the medians, red boxes indicate the interquartile range, and red whiskers indicate the range excluding outliers. Average amplitude ratios $\tilde{A_{n+\text{1}}/A_{n}}=exp(\tilde{d_{n+\text{1},n}})\;$shown on the top. Statistical significance (Wilcoxon signed-rank tests on $\tilde{d_{n+\text{1},n}}\text{≠}\text{0}$): *** p < 0.001, * p < 0.05, NS – not significant. (e) Correlations $r_{n+\text{1},n}\;$between $A_{n+\text{1}}\;$and $A_{n}$ for all whisks during basal breathing. $r_{\text{2},\text{1}}=\text{0}.\text{7}\text{1}$; $r_{\text{3},\text{2}}=\text{0}.\text{8}\text{0}$; $r_{\text{4},\text{3}}=\text{0}.\text{8}\text{6}$; $r_{\text{5},\text{4}}=\text{0}.\text{8}\text{5}$. Statistical significance ($r_{n+\text{1},n}>\text{0}$, see **Materials and Methods**): *** p < 0.001. (f) Hypothetical input (red) and output (blue) signals of a periodically driven oscillator system exhibiting a transient response to a driving pulse train at the driven oscillator’s intrinsic frequency. This schematic illustrates how amplitudes of successive oscillations following the pulse train onset are calculated. (g) Example whisking and breathing kinematics during a transition from basal breathing to sniffing. Conventions are as in Fig 1c (h) Distributions of log ratios $d_{n+\text{1},n}=\;\text{ln}\left(A_{n+\text{1}}/A_{n}\right)\;$of successive whisk amplitudes, where A represents the amplitude and n represents the nth whisk after a transition from basal breathing to sniffing, for all such transitions, as defined in the schematic in panel f. Red lines in boxes indicate the median log ratios $\tilde{d_{n+\text{1},n}}$ over all transitions to sniffing, red notches indicate 95% confidence intervals of the medians, red boxes indicate the interquartile range, and red whiskers indicate the range excluding outliers. Average amplitude ratios $\tilde{A_{n+\text{1}}/A_{n}}=exp(\tilde{d_{n+\text{1},n}})\;$shown on the top. Statistical significance (Wilcoxon signed-rank tests on $\tilde{d_{n+\text{1},n}}\text{≠}\text{0}$): *** p < 0.001, ** p < 0.01, N.S. – not significant. (i) Spectral coherence between whisking and sniffing in each 2-second sniffing segment, calculated at the *mean sniff frequency* over the segment ($\left|C_{WS}\right|$), with a time-bandwidth product of 3 and 5 tapers, versus the *mean whisk amplitude* over the corresponding segment. Filled circles represent significant coherence at p < 0.05, and open circles represent segments with non-significant coherence. (j) Mean whisk amplitude versus mean sniff frequency for each 2-second segment in panel i. Conventions are as in **panel i**. *r* represents the Pearson correlation coefficient, N.S. - not significant. (k) Phase of coherence between whisking and sniffing at the mean sniff frequency ($\varphi_{WS}$) versus the mean sniff frequency for each 2-second segment in panel i. Negative values of $\varphi_{WS}$ indicate that protraction onset leads inhalation onset. $r_{cl}$ represents the circular-linear correlation coefficient. The dashed gray line represents the regression line (no significant correlation). All other conventions are as in panel i. (l) Phase of coherence between whisking and sniffing ($\varphi_{ws}$) versus mean whisk amplitude. *** p < 0.001. The red line represents the regression line. Other conventions are as in panels i, k.

A quantitative re-analysis of simultaneously measured whisking and breathing data from rats [14,24] (Fig 1b, 1c) shows that the whisking and breathing signals have overlapping power spectra, with whisking slightly shifted to higher frequencies (Fig 2a, top). When the analysis is restricted to behavioral epochs comprised of sniffing, the whisking and breathing spectra are aligned (Fig 2a, middle) with a dominant (peak) whisking frequency of 6.1 Hz. In contrast, when only basal breathing epochs are considered, the dominant whisking frequency is higher, at 7.8 Hz, and the spectrum is broader (Fig 2a, bottom). The latter observation suggests that the typical intrinsic vIRt dominant frequency during basal breathing is offset from the typical sniffing frequency and that the observed phase-locking between sniffing and whisking [14] is likely due to the fact that breathing is resetting the vIRt oscillation on each cycle, thereby entraining the whisking rhythm (“the magnet effect” [2]).

We next evaluated the kinematics of whisking during basal breathing by comparing the relative amplitudes of successive whisking oscillation cycles following an inhalation (Fig 2b). Qualitatively, it appeared that whisks concomitant with inhalation have larger amplitude than subsequent “intervening” whisks [24] (Fig 1c). On its own, this observation is consistent with a CPG network architecture in which independent breathing and whisking oscillators both provide parallel innervation to FMNs, as well as a hierarchical model in which the breathing oscillator influences the whisking oscillator. The latter architecture is more likely in light of the additional observation that breathing introduces a phase shift in the whisking rhythm [14]. Additionally, subsequent whisks after the first inhalation-driven whisk have amplitudes that decay monotonically for the next 4 cycles [24] (Fig 2c, 2d). Together, these observations are consistent with a circuit architecture in which the breathing oscillator drives the whisking oscillator, and that aggregate spiking activity in the vIRt network that is triggered by inhalation is persistent in the network and dissipated over several oscillation cycles. If this is indeed the case, one would hypothesize that successive whisk amplitudes are correlated with one another. Our re-analysis indeed shows that for the first 4 whisks after inhalation, the amplitude of the Nth whisk is correlated with the amplitude of the N + 1st whisk (Fig 2e), and the intervening whisk amplitude reaches a steady state after 5–6 cycles (Fig 2d).

This observation also implies that if a new inhalation occurs at the preferred phase of the whisk cycle, the amplitude of the succeeding whisk should be larger than the preceding whisk, because the inhalation-driven spiking activity that is retained in the network would add constructively with new activity introduced by the inhalation. By this logic, sequential breaths that each occur at the preferred whisking phase should lead to a “ramp up” in amplitude across whisk cycles until the network reaches steady state (Fig 2f). We therefore analyzed epochs when the rats transitioned from basal breathing to sniffing (Fig 2g), and we assessed the relative amplitude of successive whisk cycles. We found that whisk amplitudes increased up to the 3^rd^ whisk after the onset of sniffing, at which point a steady-state amplitude was reached (Fig 2h). Together, these observations are consistent with the hypothesis that the vIRt network acts as a driven oscillator that exhibits entrainment [22] when driven by periodic input from a breathing CPG.

Finally, we analyzed bouts of sniffing to determine if whisking phase, amplitude, and sniffing frequency are correlated with one another. Sniffing bouts were divided into non-overlapping 2-second segments, and spectral coherence between whisking and sniffing was calculated at the *mean sniff frequency* of the segment (|$C_{WS}$|). We found that 96% of sniffing segments exhibited significant sniffing-whisking coherence at the p < 0.05 level (Fig 2i), suggesting strong phase-locking that is consistent with previous observations [3,14,31]. We found that whisking over a broad range of amplitudes, with the mean whisk amplitude per segment ranging from 10 to 80°, could be observed across the full range of sniffing frequencies (> 5 Hz), and that the mean whisk amplitude was not significantly correlated with the mean sniff frequency (r = 0.05, p = 0.27) (Fig 2j). Across all sniffing frequencies, the mean phase of whisking-sniffing coherence at the sniffing frequency ($\varphi_{\text{WS}}$) was -0.84 ± 0.37 radians (circular mean ± circular std. dev. [32]). $\varphi_{\text{WS}}$ did not increase significantly with the mean sniff frequency (circular-linear correlation [32]; r = 0.11, p = 0.075) (Fig 2k), suggesting the possibility that the whisking and sniffing oscillators’ frequencies are co-modulated during natural whisking behavior to achieve a consistent phase relationship. Intriguingly, $\varphi_{\text{WS}}$ exhibited a significant correlation with the mean whisk amplitude (circular-linear correlation r = 0.41, p ≈ 0 (below numerical precision)), with a phase change of -0.57 radians over the full range of whisk amplitudes (10–80°) (Fig 2l). This systematic phase shift with amplitude is consistent with the hypothesis that deviations in the whisking oscillator intrinsic frequency within the 1:1 mode modulate whisking amplitude.

### Adaptive exponential integrate-and-fire model of the whisking CPG

**Fig 3:**
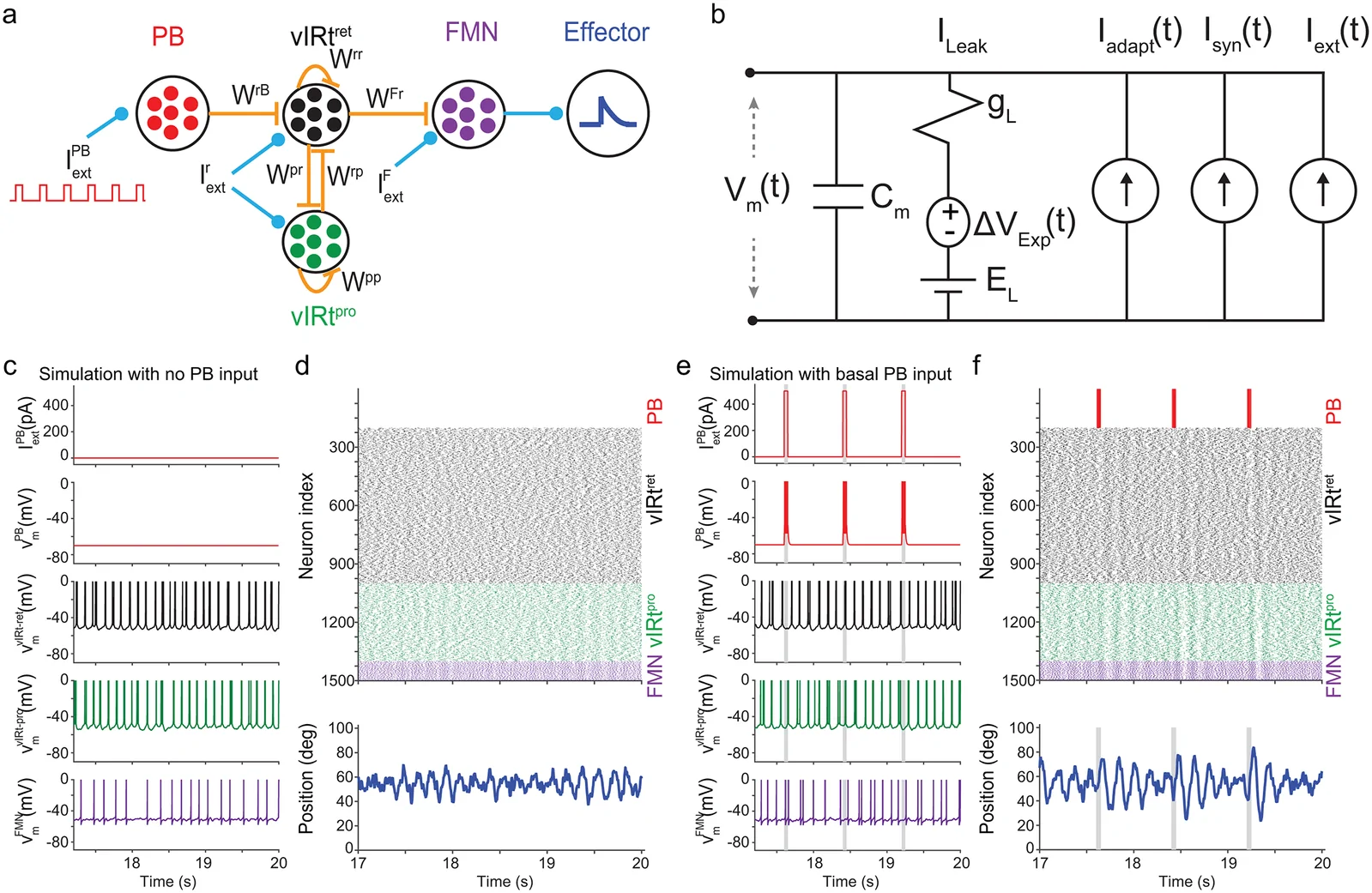
Adaptive exponential integrate-and-fire neuronal network model of the whisking CPG. (a) Schematic of a neural network model of the vibrissa intermediate reticular formation (vIRt) protraction and retraction neuron pools (${\text{vIRt}}^{\text{pro}}$ and ${\text{vIRt}}^{\text{ret}}$, green and black, respectively), preBӧtzinger complex inhalation pool (PB, red), facial motor neuron pool (FMN, purple) and end effector model (blue). Inhibitory connections are shown in yellow, and excitatory input currents are shown in light blue. Abbreviations: external current inputs to PB, vIRt, FMN neuron pools: $I_{ext}^{PB}$, $I_{ext}^{r}$, $I_{ext}^{F}$, respectively; synaptic current connectivity matrices PB to ${\text{vIRt}}^{\text{ret}}$ ($W^{rB}$), ${\text{vIRt}}^{\text{ret}}$ to ${\text{vIRt}}^{\text{ret}}$ ($W^{rr}$), ${\text{vIRt}}^{\text{pro}}$ to ${\text{vIRt}}^{\text{pro}}$ ($W^{pp}$), ${\text{vIRt}}^{\text{pro}}$ to ${\text{vIRt}}^{\text{ret}}$ ($W^{rp}$), ${\text{vIRt}}^{\text{ret}}$ to ${\text{vIRt}}^{\text{pro}}$ ($W^{pr}$), ${\text{vIRt}}^{\text{ret}}$ to FMN ($W^{Fr}$) (see **Materials and Methods**). (b) Electrical equivalent circuit model of each neuron in the network. Electrical quantities are denoted as: membrane potential ($V_{m}$), membrane capacitance ($C_{m}$), leak conductance ($g_{L}$), leak current ($I_{L}$), leak potential ($E_{L}$), time-dependent exponential term ($\Delta V_{Exp}(t)$), adaptation current ($I_{adapt}(t)$), synaptic current ($I_{syn}(t)$), external input current ($I_{ext}(t)$). According to the adaptive exponential integrate-and-fire model, a spike is emitted when $V_{m}$ reaches threshold $V_{T}$ and immediately reset to $V_{reset}$*.* Each spike results in a contribution to $I_{syn}$ in the post-synaptic neurons (see **Materials and Methods**). (c) Example segment of a simulation of the network in **panel a** with $I_{ext}^{PB}=\text{0}$. Plots of $I_{ext}^{PB}$ (red), and examples of membrane potential traces in individual neurons from each pool $V_{m}^{PB}$ (red), $V_{m}^{vIRt-ret}$ (black), $V_{m}^{vIRt-pro}$ (green), and $V_{m}^{FMN}$ (purple). (d) Example segment of a simulation of the network in **panel a** with $I_{ext}^{PB}=\text{0}$. (top) Raster plots of spike times from each neuron in PB (no spikes), ${\text{vIRt}}^{\text{ret}}$ (black), ${\text{vIRt}}^{\text{pro}}$ (green), and FMN (purple) pools. (bottom) Effector position (blue) obtained by convolving the aggregate spiking activity of all FMN neurons with an alpha function with a time constant of $\tau_{\alpha }$. (e) Example segment of a simulation of the network in **panel a** with $I_{ext}^{PB}\;$as a pulse train with pulse duration 50ms, amplitude 500pA, and frequency 1.25 Hz. Plots of $I_{ext}^{PB}$ (red), and examples of membrane potential traces in individual neurons from each pool. Conventions are as in panel c. (f) Example segment of a simulation of the network in **panel a** with $I_{ext}^{PB}\;$as a pulse train as in panel e. (top) Raster plots of spike times from each neuron in each pool. Gray bars correspond to $I_{ext}^{PB}\;$pulse times. (bottom) Effector position (blue) from the same simulation. Spike times from PB model neurons are shown in red. Gray bars correspond to $I_{ext}^{PB}\;$pulse times. Other conventions are as in panel d.

To assess whether a network of neurons connected consistent with physiological observations of the vIRt [14,15,25] can produce oscillations with the kinematic signatures and phase dynamics of entrainment in natural rat whisking (Fig 2), we constructed a neuronal network model of the vIRt whisking oscillator with adaptive exponential integrate-and-fire model neurons using current-based synapses [33] (Fig 3a, 3b). This model is an adaptation of the model network structure described by Golomb et al. [24], which uses conductance-based model neurons. This simplification enables us to demonstrate that the model oscillator’s coupling phenomena we evaluate do not require state-dependent synaptic gain (shunting) or the biophysical properties of specific ion channels, and that they can instead arise from network-level dynamics. Such a demonstration is relevant given the paucity of electrophysiological data constraining parameters at the channel or synapse levels in the vIRt, though we do not exclude that specific ion-channel biophysical properties can further shape these dynamics in vivo. The model has the added advantage of being computationally efficient for exploration of large parameter spaces for both the present study and potentially in future studies. Briefly, the model includes four pools of neurons, PB inhibitory inhalation neurons, ${\text{vIRt}}^{\text{ret}}$ inhibitory neurons, ${\text{vIRt}}^{\text{pro}}$ inhibitory neurons, and facial motor neurons (FMNs). For this model, we made rough estimates of the number of neurons in each pool (rounded to nearest 100) based on previous experimental observations: 200 inhibitory inspiratory PB neurons [34–36], 800 inhibitory ${\text{vIRt}}^{\text{ret}}$ neurons, 400 inhibitory ${\text{vIRt}}^{\text{pro}}$ neurons [14,25,37], and 100 vibrissa FMNs [38] (see **Materials and Methods** for calculations).

Sparse, random subsets of the neurons in the ${\text{vIRt}}^{\text{ret}}$ (2/3 of total vIRt neurons) and ${\text{vIRt}}^{\text{pro}}$ (1/3 of total vIRt neurons) pools synapse onto one another and carry inhibitory synaptic currents of a defined amplitude for each source and target neuron pool. This asymmetric ${\text{vIRt}}^{\text{ret}}$ to ${\text{vIRt}}^{\text{pro}}$ neuron ratio of 2:1 is selected based on evidence from *in vivo* recordings [14,25]. ${\text{ vIRt}}^{\text{ret}}$ neurons additionally receive inhibitory synaptic input from PB neurons, and inhibit FMNs, also with current amplitudes defined for each source and target neuron pool (see **Materials and Methods**). Synaptic currents are injected into the postsynaptic neuron upon a spike in the presynaptic neuron and decay with a time constant of $\tau_{syn}$, estimated to be approximately 10 ms for inhibitory gamma-aminobutyric acid type A (GABA_A_) and glycine receptors. Each population receives external excitatory currents *I*_*ext*_, which can be constant or time-varying. The aggregate spiking activity of the pool of FMNs is translated into an end-organ effector (vibrissa) movement signal by convolving with an alpha function with a time constant of $\tau_{\alpha }$ = 10 ms and amplitude of 2°, based on the previous estimates of the transfer function between vibrissa FMN spikes and movement [38], and assuming linearity. This assumption of a linear FMN to movement transfer function enables us to model and evaluate the effects of interactions between neuronal oscillators without the confound of potentially nonlinear biomechanics.

Finally, as in [24], our model vIRt neurons exhibit short-term adaptation, which is the mechanism underlying the phase transitions in the model half-center oscillator network that renders it rhythmogenic. In the adaptive exponential integrate-and-fire neurons the adaptation is given by subthreshold and spike rate adaptation parameters, *a, b,* and $\tau_{w}$ [39]. This adaptation mechanism is also responsible for the ability of the model network to retain activity of previously induced spiking activity across oscillation cycles. Tables of the neuron and network parameters described above are provided in **Materials and Methods**. With these network parameters and tonic excitatory input currents applied to each of the neurons in the ${\text{vIRt}}^{\text{pro}}$, ${\text{vIRt}}^{\text{ret}}$, and FMN pools but not the PB pool, a simulation of the vIRt intrinsic dynamics results in rhythmic oscillations of the end effector (Fig 3c, 3d) [24]. In a supplementary analysis, we evaluated the sensitivity of oscillations in this model vIRt/FMN network with no external periodic drive to intra-vIRt synaptic current amplitudes (${\text{W}}^{\text{rr}}$, ${\text{W}}^{\text{pp}}$, ${\text{W}}^{\text{rp}}$, ${\text{W}}^{\text{pr}}$) (Figs 3a, S1a). We systematically varied these values and calculated the steady-state *intrinsic frequency* (S1a Fig, top left), *root-mean-square (RMS) amplitude* (bottom left), *set-point* (top right), and *spectral peak signal-to-noise ratio (SNR)* (bottom right) of the resulting effector output signal (see **Materials and Methods** for definitions). This analysis reveals that adaptation in ${\text{vIRt}}^{\text{ret}}$ neurons alone, without any input from ${\text{vIRt}}^{\text{pro}}$, can produce oscillations at a fixed frequency for the given set of adaptation parameters. This result is consistent with physiological observations of kainic acid-induced whisking in urethane-anesthetized rats, in which rhythmic firing in vIRt neurons was locked to retraction, but not protraction, and induced whisking was desynchronized from breathing [25]. In contrast, under ketamine/xylazine anesthesia and in alert rats and mice, both ${\text{vIRt}}^{\text{ret}}$ and ${\text{vIRt}}^{\text{pro}}\;$neuronal populations are observed [14,15,25]. Inter-connections between ${\text{vIRt}}^{\text{ret}}$ and ${\text{vIRt}}^{\text{pro}}$ neuron populations in our model network provide variability in intrinsic frequency, RMS amplitude, and set-point that depends on the strength of the inter-pool currents ($W^{rp}$, $W^{pr}$) relative to the intra-pool currents ($W^{rr}$, $W^{pp}$) (S1 Fig). We therefore selected values of inter-pool synaptic currents that slightly exceeded intra-pool currents for subsequent analysis, consistent with a previous computational model [24] (see **Materials and Methods**). When this same network is stimulated with 50-ms square pulses of input current to the neurons in the PB pool, we qualitatively observe larger amplitude PB-driven oscillations which decay over successive oscillation cycles (Fig 3e, 3f). This model behavior is qualitatively similar to rat whisking at basal breathing frequencies (Fig 2c, 2d).

### Model whisking CPG network responses to periodic PB stimulation at varying frequencies

**Fig 4:**
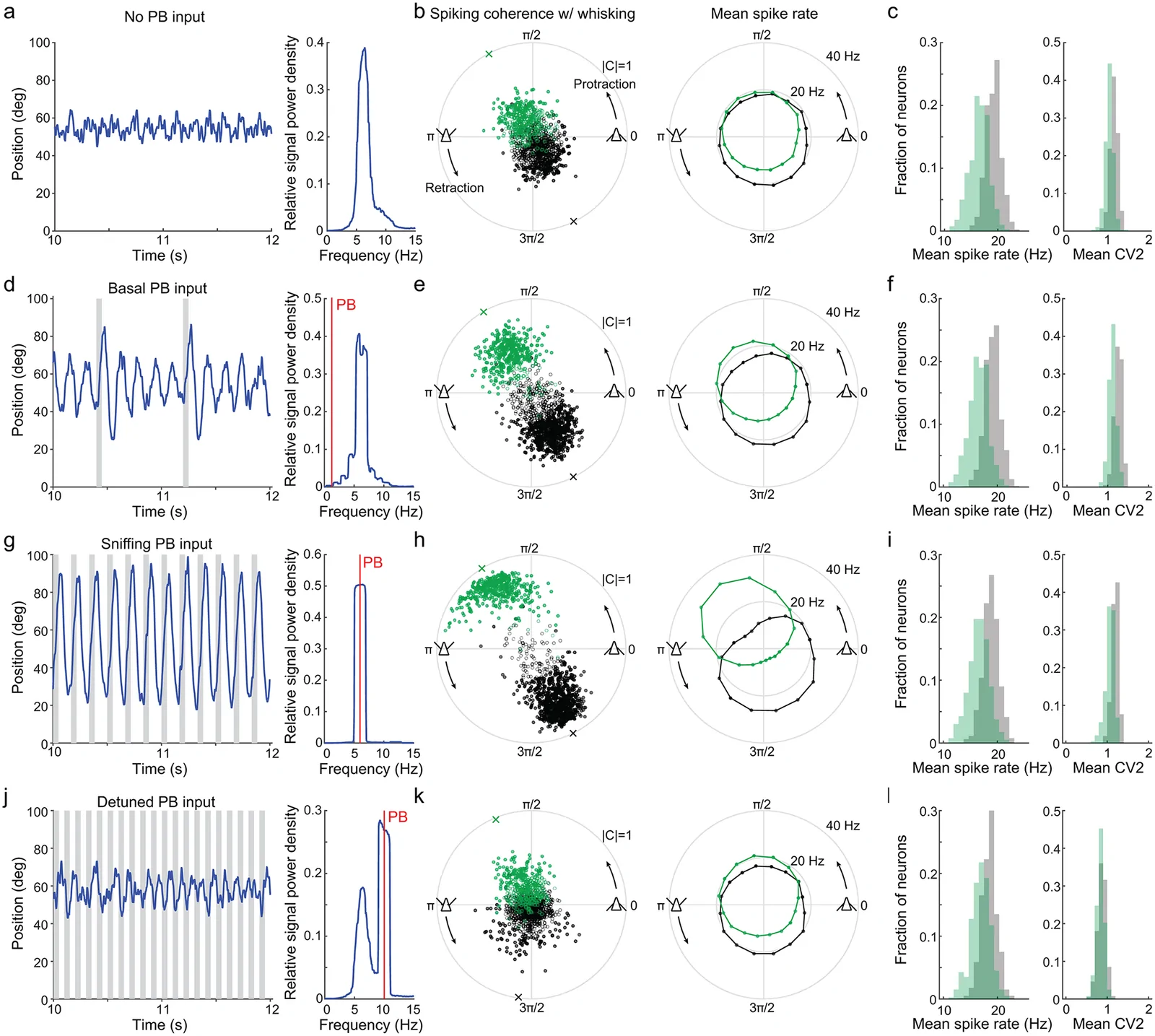
Model whisking CPG network with periodic drive at different frequencies. (a) (left) Example segment of the effector signal in a simulation of the network in Fig 3a with $I_{ext}^{PB}=\text{0}$, as in Fig 3d, bottom. (right) Power spectrum of the effector signal at steady state (last 15 seconds of 30-second runs) to the left. (b) (left) Magnitude (radial axis) and phase (tangential axis) of the coherence of each ${\text{vIRt}}^{\text{pro}}$ (green circles) and ${\text{vIRt}}^{\text{ret}}$ (black circles) model neuron with the effector position signal at the frequency with maximum power, 6.4 Hz, in **panel a, right**. Filled circles represent neurons whose coherence is statistically significant at p < 0.05, and open circles are not significant. (right) Mean spike rate (radial axis) in each phase bin (tangential axis) over all ${\text{vIRt}}^{\text{pro}}$ neurons (green) and ${\text{vIRt}}^{\text{ret}}$ neurons (black) (c) (left) Histogram of mean spike rates for each model neuron over the full simulation for ${\text{vIRt}}^{\text{pro}}$ (green bars) and ${\text{vIRt}}^{\text{ret}}$ (black bars) populations. (right) Histograms of mean CV2 values (see **Materials and Methods**) for each model neuron over the simulation for ${\text{vIRt}}^{\text{pro}}$ (green bars) and ${\text{vIRt}}^{\text{ret}}$ (black bars) populations. (d-f) Results of simulation of the network in Fig 3a with $I_{ext}^{PB}\;$as a pulse train with pulse duration 50 ms, amplitude 500 pA, and frequency 1.25 Hz. Gray bars in **panel d, left** correspond to $I_{ext}^{PB}\;$pulse times. Red line in **panel d, right** corresponds to the pulse train frequency. Other conventions are as in **panels a-c. (g-i)** Results of simulation of the network in Fig 3a with $I_{ext}^{PB}\;$as a pulse train with pulse duration 50 ms, amplitude 500 pA, and frequency 6 Hz. Conventions are as in panels d-f. (j-l) Results of simulation of the network in Fig 3a with $I_{ext}^{PB}\;$as a pulse train with pulse duration 50 ms, amplitude 500 pA, and frequency 10 Hz. Conventions are as in **panels d-f.**

If the vIRt whisking oscillator circuit were to exhibit frequency-selective amplification when driven periodically by breathing, then the amplitude of the inhalation-driven whisks, i.e., the first whisk after each inhalation, would vary systematically with the breathing rate and exhibit a peak amplitude at the vIRt intrinsic frequency. Therefore, to quantitatively assess how vIRt whisking oscillator network dynamics may vary with breathing frequency, we simulated the vIRt model network described above (Fig 3) and examined the spectral properties of the steady-state effector output signal and the corresponding spike train statistics of the vIRt neurons. First, we find that when PB neurons are not spiking, a rhythmic effector oscillation with a single spectral peak at 6.4 Hz is observed (Figs 3d, 4a). The spectral coherence between the aggregate spiking activity of the ${\text{vIRt}}^{\text{ret}}$ neurons and the effector position at its dominant frequency is 0.995 at a phase of 5.16 rad, or just after mid-retraction. Correspondingly, the spectral coherence of the aggregate spiking activity of the ${\text{vIRt}}^{\text{pro}}$ neurons with the effector signal is 0.994 at a phase of 2.06 rad, or just after mid-protraction (Fig 4b, left, x’s). There is considerable spread in the coherence magnitudes of individual neurons from approximately 0 to 0.6 (Fig 4b, left, dots). The mean spike rate per neuron within each pool as a function of phase in the effector cycle, e.g., the neuronal “phase tuning curve” [18], shows a slight bias towards the mid-retraction phase for ${\text{vIRt}}^{\text{ret}}$ neurons, and a slight bias towards the mid-protraction phase for ${\text{vIRt}}^{\text{pro}}$ neurons (Fig 4b, right). Overall, the mean spike rates range between ~15 and 25 Hz for ${\text{vIRt}}^{\text{ret}}$ neurons, and ~10–20 Hz for ${\text{vIRt}}^{\text{pro}}$ neurons (Fig 4c, left). The CV2, a widely used metric of spike timing variability calculated between adjacent pairs of spikes [40], is clustered near 1 for both pools (Fig 4c, right). This relatively high CV2 is consistent with previous single-unit recordings of vIRt neurons in both alert, whisking mice and kainic-acid induced whisking rats [24], and is indicative of fluctuation-driven spiking [41]. In a previous modeling study, this relatively high CV2 is due to intra-pool inhibition being almost as strong as inter-pool inhibition [24], and this is also the case in the present model (Figs 3a, S1).

Next, we simulated the responses of this model vIRt/FMN network to pulse trains of input current to PB neurons at varying frequencies (Fig 4d-4l). During low-frequency stimulation (1.25 Hz) that simulates basal breathing, the dominant frequency of the effector signal (5.8 Hz) remained close to the intrinsic frequency (6.4 Hz) (Fig 4d). The spiking coherence magnitudes of individual neurons with the effector output signal increased, with fewer neurons exhibiting low coherence values (Fig 4e). The phase tuning curves of ${\text{vIRt}}^{\text{ret}}$ and ${\text{vIRt}}^{\text{pro}}$ neuron pools separated more than in the undriven case (Fig 4e vs. 4b), yet the overall spike rates and CV2 values did not change appreciably (Fig 4f vs. 4c). With PB stimulation at 6 Hz, approximately the dominant intrinsic vIRt frequency in the model, the amplitude and spectral purity of the effector output signal increased relative to the case of basal breathing frequency stimulation (Fig 4g vs. 4d), and the dominant frequency is at the intrinsic frequency (6.4 Hz), demonstrating frequency-selective amplification. The magnitude of coherence of the individual neurons increased further, and the ${\text{vIRt}}^{\text{ret}}$ and ${\text{vIRt}}^{\text{pro}}$ phase tuning curves separated further (Fig 4h vs. 4e), while the mean spike rates and CV2 values remained relatively consistent (Fig 4i). These observations suggest that oscillations are produced by shifting the timing of spikes rather than drastically increasing the overall spike rates of the neurons from quiescence, consistent with experimental results [15]. Finally, stimulation at 10 Hz, which is greater than the intrinsic oscillation frequency of the vIRt network, resulted in an output signal with a lower amplitude and a bimodal power spectrum (Fig 4j), with the dominant frequency close to the drive frequency (9.3 Hz). Here, the magnitude of coherence values of vIRt units at the dominant frequency decreased as compared to the case of the 6 Hz drive frequency (Fig 4k vs. 4h) with similar spike train statistics (Fig 4l vs. 4i). These observations indicate that the oscillation amplitude is sensitive to the drive frequency, consistent with an entrained oscillator (Fig 4a, 4d, 4g, 4j) [22].

**Fig 5:**
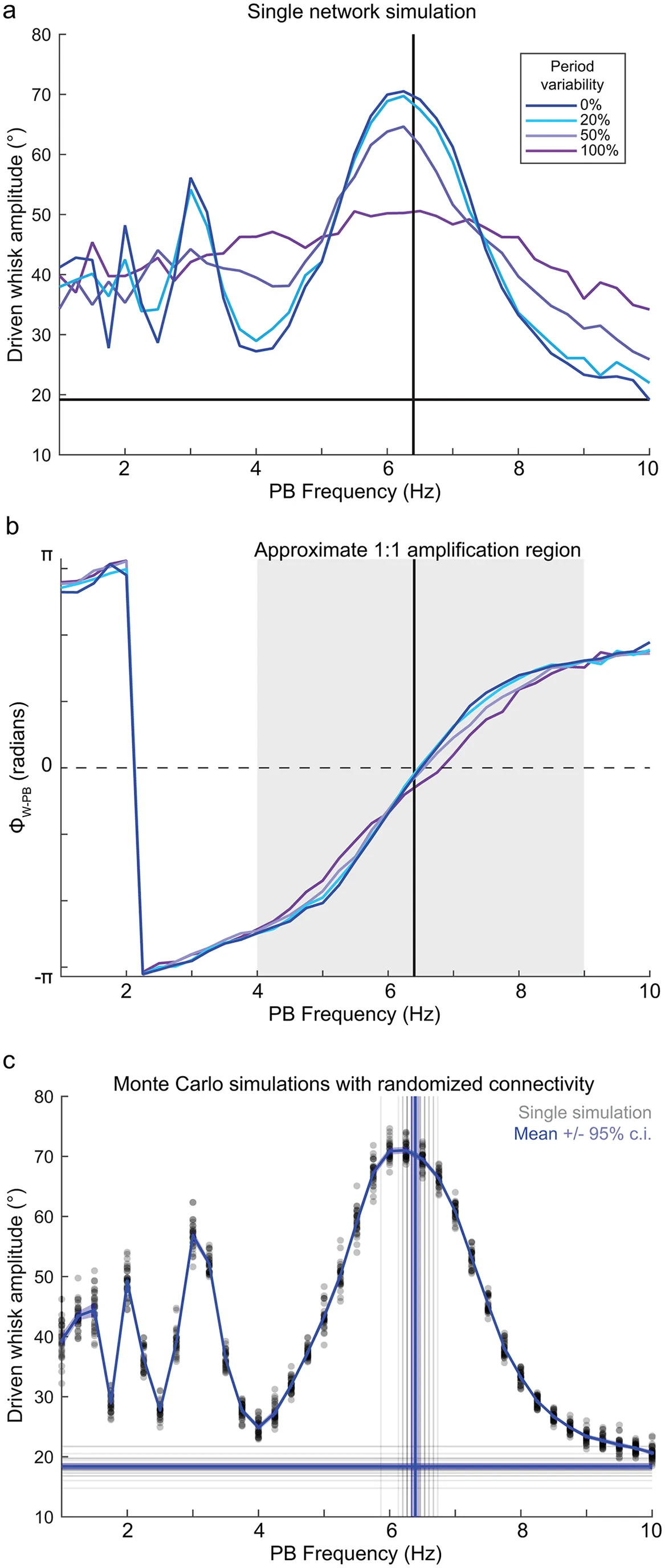
Model whisking CPG network with periodic drive exhibits frequency-selective amplification. (a) Plot of the mean amplitude of the first whisk following each $I_{ext}^{PB}$ pulse at steady state (*driven whisk amplitude*) for simulations of the network in Fig 3a at $I_{ext}^{PB}$ pulse frequencies ranging from 1-10 Hz. Random variability in each inter-pulse period is 0% of the period duration (blue trace), 20% (light blue trace), 50% (violet trace) and 100% (purple trace) (see **Materials and Methods**). The horizontal and vertical black bars represent the mean *intrinsic amplitude* and the *intrinsic frequency*, respectively, of simulations in which $I_{ext}^{PB}$ = 0. (b) Plot of the phase of coherence between PB pulse onset times and the effector signal, calculated at the PB pulse frequency ($\varphi_{W-PB}$), vs. PB pulse frequency. Negative values of $\varphi_{W-PB}$ indicate that protraction onset leads inhalation onset. The vertical black bar represents the intrinsic frequency of simulations in which $I_{ext}^{PB}$ = 0. Colors represent different period variability values, as in panel a. The approximate PB pulse frequency range where the effector signal is amplified in the 1:1 regime is shown in gray (c) Plot of the *driven whisk amplitude* for simulations of the network in Fig 3a at $I_{ext}^{PB}$ pulse frequencies ranging from 1-10 Hz. Each gray dot represents a simulation with a different random instantiation of the connectivity matrices $W^{rB}$, $W^{rr}$, $W^{pp}$, $W^{rp}$, $W^{pr}$, $W^{Fr}$ (Monte Carlo simulations). The gray horizontal and vertical lines represent the mean intrinsic amplitude and the intrinsic frequency, respectively, of whisking at steady state for simulations of the network in Fig 3a with $I_{ext}^{PB}$= 0. Again, each line represents a different random instantiation of the connectivity matrices $W^{rB}$, $W^{rr}$, $W^{pp}$, $W^{rp}$, $W^{pr}$, $W^{Fr}$. The blue trace represents the mean of the 30 instantiations, and the light blue shaded area is the 95% bootstrap confidence band of the mean. The blue horizontal and vertical lines represent the mean vIRt intrinsic amplitude and frequency, respectively, of the 30 random instantiations of the connectivity matrices. The light blue shaded vertical and horizontal bands represent 95% bootstrap confidence intervals of the mean.

To quantify the effector amplitude dependence on breathing drive frequency, we simulated the activity of the model vIRt/FMN whisking CPG in response to PB pulse frequencies ranging from 1-10 Hz, and we calculated the mean amplitude of the first whisk oscillation cycle after each pulse (*driven whisk amplitude*) (Fig 5a, blue trace). For direct comparisons between whisk amplitudes in the driven and undriven cases, we define the *intrinsic amplitude* (black horizontal line) as the mean amplitude of the first oscillation cycle after PB “pseudo pulses” (with amplitude 0pA; see **Materials and Methods** for definitions). We observe a peak in the driven whisk amplitude close to the intrinsic frequency of the vIRt network (black vertical line), corresponding to the 1:1 mode, and lower amplitude peaks at integer ratios of this frequency to the drive frequency (2:1, 3:1, etc). At all drive frequencies, the mean amplitude of the first PB driven oscillation cycle is greater than the amplitude of the intrinsic vIRt oscillations, due to the spiking induced by the external drive to the vIRt network. The observed local peaks at N:1 output to drive frequency ratios are consistent with the notion that because each PB pulse resets the phase of the whisking oscillator, the subsequent pulse occurs at the same constructive phase of the whisk cycle as when driven at the intrinsic vIRt frequency, except on every Nth cycle, where N is an integer greater than 1. In reality, the breathing frequency is not stationary, so in order to determine how variability in the breathing frequency affects the amplification, we systematically jittered the PB inter-pulse period at each frequency proportionally to its duration (see **Materials and Methods**). This variation had the effect of “flattening out” the N:1 peaks, because the additional variability constitutes a larger proportion of the vIRt intrinsic period at lower stimulation frequencies (Fig 5a, light blue lines). The observation that the N:1 peaks are smaller in amplitude than the 1:1 peak near the vIRt intrinsic frequency, at all levels of period variability, suggests there is an additional amplification at the intrinsic frequency due to network activity that reverberates and decays across oscillation cycles, as expected from a system exhibiting entrainment [22].

We next assessed the phase relationship between simulated PB pulse trains and whisking over 1–10 Hz PB pulse frequencies for the network instantiation used in Figs 3, 4, 5a. We observed an approximately linear whisking-to-PB phase shift with PB pulse frequency across the 1:1 amplification frequency range (roughly 4–9 Hz) (Fig 5b). A supplementary analysis demonstrates that increasing the synaptic drive from PB to ${\text{vIRt}}^{\text{ret}}$ neurons ($W^{rB}$, Figs 3a, S2) widens the 1:1 amplification frequency range and decreases the slope of the phase shift across frequencies. These observations are also expected based on theoretical predictions for a driven nonlinear oscillator with a constant intrinsic frequency, in which the width of the 1:1 amplification regime depends on coupling strength [22]. In contrast to this linear phase shift with PB pulse frequency observed in the model (Fig 5b), rats appear to whisk with a phase relationship to sniffing that is uncorrelated with sniffing frequency (Fig 2k). These observations are consistent with the detuning hypothesis: essentially, the vIRt intrinsic frequency is actively modulated to track the sniffing frequency with deviations that correspond to amplitude modulations (Fig 2j-2l). These observations indicate that the rat could actively control the intrinsic frequency of the vIRt to track the sniffing frequency, and that it can modulate this intrinsic frequency to select the desired whisking amplitude.

Finally, we note that our preceding simulations (Figs 3, 4, 5a, 5b) are the result of a vIRt network with fixed connectivity. That is, the identities of which neurons within the ${\text{vIRt}}^{\text{ret}}$ population receive synaptic input from PB neurons and ${\text{vIRt}}^{\text{pro}}$ neurons, and which project to ${\text{vIRt}}^{\text{pro}}$ neurons and facial motor neurons are chosen at random. To determine how different random instantiations of these connectivity patterns affect the amplification as a function of PB frequency, we ran Monte Carlo simulations with different random instantiations of the connectivity matrices (Fig 5c). The results of each of 30 instantiations are shown along with the corresponding dominant vIRt intrinsic frequencies and amplitudes, and the mean and 95% confidence interval across instantiations are shown in blue. The results of these Monte Carlo simulations indicate that the amplitude amplification peak near the vIRt intrinsic frequency is highly robust across different random connectivity instantiations in the PB/vIRt/FMN network model.

### Dependence of vIRt intrinsic frequency on input current statistics

**Fig 6:**
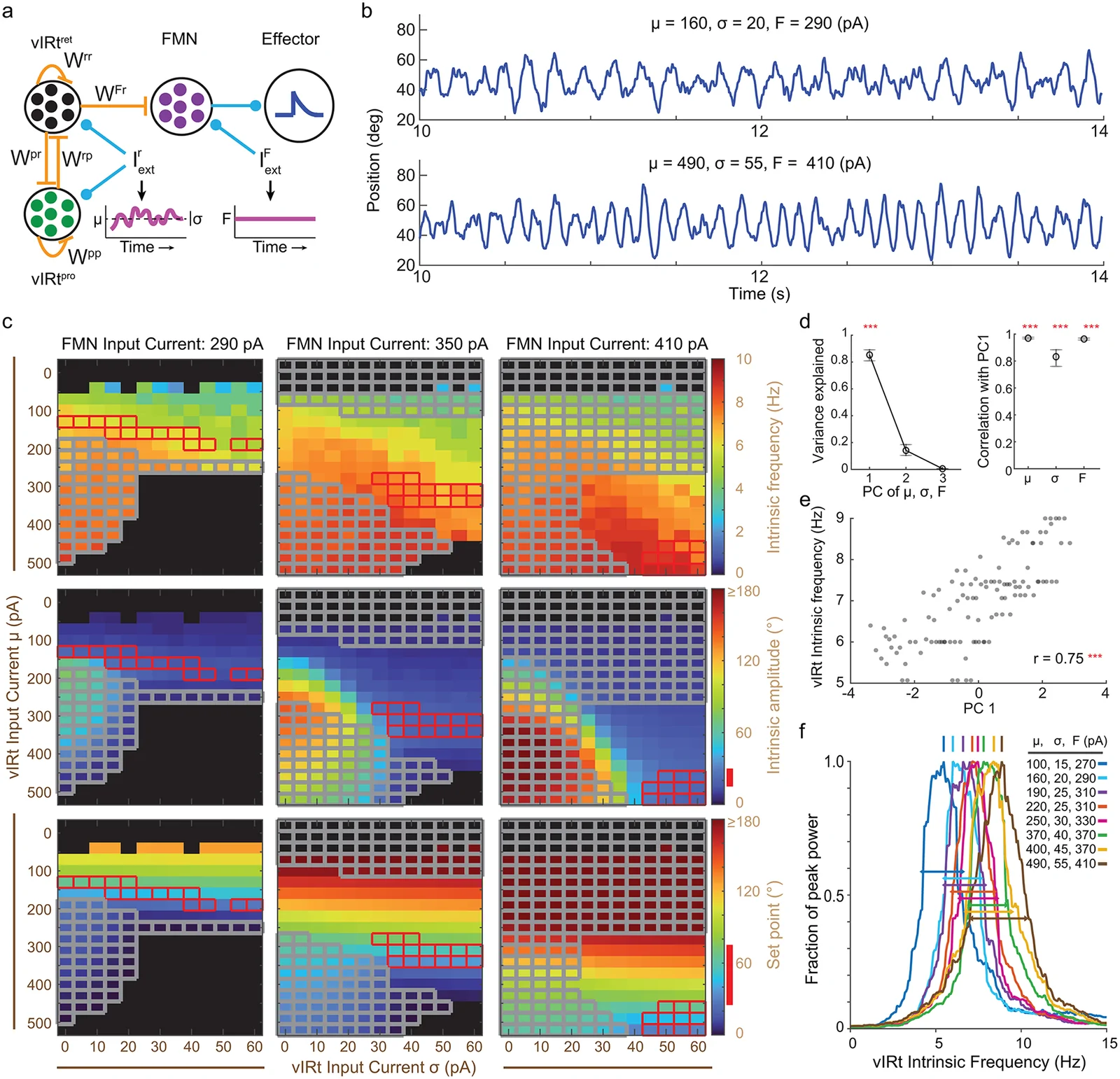
Model whisking CPG network with no periodic drive and noisy external input currents can produce oscillations at a range of intrinsic frequencies. (a) Schematic of neural network model of the vibrissa intermediate reticular formation (vIRt) with time-varying external input current to ${\text{vIRt}}^{\text{pro}}$ and ${\text{vIRt}}^{\text{ret}}$ neuron pools, constant external input current to FMNs (magenta), and no PB input to the ${\text{vIRt}}^{\text{ret}}$ neuron pool. Conventions are as in Fig 3a. The external input current to ${\text{vIRt}}^{\text{pro}}$ and ${\text{vIRt}}^{\text{ret}}$ is implemented as an Ornstein-Uhlenbeck process [42] with a mean value *µ*, a standard deviation *σ*, and a fixed time constant of 5 ms. The external input current to FMN is implemented as a constant level *F*. (b) Example segments of two different simulations with different values of *µ*, *σ*, *F* resulting in different dominant intrinsic oscillation frequencies of 6.0 Hz (top) and 8.9 Hz (bottom). (c) (top row) Heatmaps of the intrinsic oscillation frequency for simulations across a range of values for *σ* (x-axis) and *µ* (y-axis). Each of the 3 heatmaps represents a different representative value of *F*: 290 pA (left), 350 pA (middle), 410 pA (right). Color represents the frequency according to the scale to the right. (middle row) Heatmaps of oscillation intrinsic amplitudes for the simulations represented above. (bottom row) Heatmaps of oscillation set-points for the simulations represented above. Red boxes indicate simulations that result in intrinsic amplitudes between 15 and 30° and set-points between 25 and 75°. Red vertical bars indicate the target intrinsic amplitude and set-point ranges. Simulations producing oscillation intrinsic amplitudes <5° are shown in black. Simulations producing oscillations whose range (set-point ± half-amplitude) extends below or above the approximate physical limits of 0 and 180°, respectively, (see Fig 1b) are annotated with gray boxes. (d) Principal component analysis of the vIRt and FMN input current parameter space of *µ*, *σ*, *F* values resulting in effector oscillations within the target amplitude and set-point range in **panel c** (see **Materials and Methods**). (left) Fraction of variance explained by each principal component (PC). *** p < 0.001, permutation test versus the null hypothesis that the variance explained is not greater than chance. (right) Correlation coefficients of each of *µ*, *σ*, *F* with PC1. Error bars represent 95% bootstrap confidence intervals. *** p < 0.001, permutation test versus the null hypothesis of no correlation. (e) Plot of the values of the intrinsic frequency corresponding to each value of PC1 from the analysis in **panel d.** *r* denotes the correlation coefficient. *** p < 0.001, permutation test versus the null hypothesis of no correlation. (f) Power spectra normalized to the peak value for representative simulations in **panel c** that result in oscillation intrinsic frequencies that span 5-9 Hz with intrinsic amplitudes between 15 and 30°. and set-points between 25 and 75°. The *σ*, *µ*, *F* values for each spectrum are shown in the legend. Double arrows indicate the full width at half maximum. Tick marks represent the intrinsic frequency.

As noted above, behavioral evidence that the whisking oscillator circuit entrains to the breathing oscillator during sniffing implies that if the animal can volitionally control the frequency of either or both oscillators, the amplitude of whisking would be dependent on the frequency mismatch (Fig 5a, 5c). The ability to control the intrinsic frequency of the vIRt oscillator would give the most behavioral flexibility, as the animal would be able to tune the whisking amplitude at any arbitrary breathing frequency. In our model, the frequency of the vIRt oscillations is dependent on both the input current to vIRt neurons, and the strength of the inter-pool synaptic currents (${\text{vIRt}}^{\text{pro}}$ to and from ${\text{vIRt}}^{\text{ret}}$) relative to the intra-pool synaptic currents [24] (Figs 3a, S1). Given that the amplitude of whisking can be modulated on the relatively fast time scale of approximately 1 s [18], the most parsimonious mechanism to generate vIRt frequency changes on that time scale would likely be to alter the input currents to vIRt, which could come from changing presynaptic inputs from other brain areas and/or cell types. Therefore, to explore the range of vIRt intrinsic dynamics that can be achieved with a fixed connectivity network, we simulated noisy input current waveforms that were applied symmetrically with the same statistics (but not identically) to the neurons in both the ${\text{vIRt}}^{\text{pro}}$ and ${\text{vIRt}}^{\text{ret}}$ neuron pools (see **Materials and Methods**). The input current dynamics were simulated to follow an Ornstein-Uhlenbeck process [42] with a mean value *µ*, a standard deviation *σ*, and a fixed time constant of 5 ms. Such a process is an instantiation of low-pass filtered Gaussian white noise and is well-established for modelling synaptic noise [42–44]. A tonic current of *F* was applied to the pool of facial motor neurons (Fig 6a). For different selections of the current parameters *µ, σ*, and *F*, we could simulate effector movements driven by vIRt intrinsic dynamics, i.e., in the absence of inputs from PB, with varying frequencies and similar amplitudes (Fig 6a, 6b).

To systematically examine how combinations of *µ, σ*, and *F* affect the whisking effector kinematics, we systematically varied these values and calculated the steady-state intrinsic frequency (Fig 6c, top), intrinsic amplitude (middle), and set-point (bottom) of the resulting whisking effector output signal. Increasing *µ*, the mean input current to vIRt neurons, with no noise (*σ* = 0 pA) increases the intrinsic frequency, but also affects the intrinsic amplitude and set-point. We therefore identified sets of parameters *µ, σ*, and *F* that produce outputs with dominant frequencies between 5 and 9 Hz, while maintaining intrinsic amplitudes and set-points in similar ranges as the baseline test case in Fig 3d; that is, intrinsic amplitudes between 15 and 30° and set-points between 25 and 75° (Fig 6c, red squares). A principal component analysis of the sets of values for the parameters *µ, σ*, and *F* that result in oscillations with intrinsic amplitudes and set-points in these ranges shows that a single principal component (PC1) accounts for over 80% of the variance in the relevant parameter space (Fig 6d, left), and that the variables, *µ, σ*, and *F*, are all positively correlated with PC1 with coefficients of 0.97, 0.83, and 0.96, respectively (Fig 6d, right). Additionally, PC1 is also positively correlated with the intrinsic frequency of the oscillations (r = 0.75, p < 0.001), indicating that, in general, increasing all three parameters, *µ, σ*, and *F*, together results in oscillations of increasing frequency while maintaining amplitude and set-point within the baseline range (Fig 6e). These observations suggest that the rat could, in principle, control the intrinsic frequency of vIRt-driven whisking independently of the intrinsic amplitude and set-point by increasing both the mean level (*µ*) and the amplitude of broad-band fluctuations in the input current to vIRt neurons (*σ*), while simultaneously increasing the depolarization of facial motor neurons (*F*). Input currents whose broad-band noise level increases independently of the mean are characteristic of neurons that receive simultaneous inputs from excitatory and inhibitory presynaptic populations [44]. Therefore, a parsimonious mechanism that could theoretically enable control over the vIRt frequency would be if it were to receive inputs from a single upstream brain region that provides approximately balanced excitatory and inhibitory projections to both the ${\text{vIRt}}^{\text{pro}}$ and ${\text{vIRt}}^{\text{ret}}$ populations together, along with a separate or parallel source of excitatory inputs to the FMNs.

### Relationship between whisking amplitude and 𝐯𝐈𝐑𝐭/𝐏𝐁 frequency mismatch

Given that vIRt intrinsic effector oscillations with similar amplitudes and set-points at different frequencies can be achieved by varying the input currents to vIRt and FMNs (Fig 6a-6e), we next sought to determine how the vIRt network operating at different intrinsic frequencies responds to driving input from PB across the ethological range of breathing frequencies. By inspection, we selected eight example sets of input current parameters *µ, σ*, and *F* with monotonically increasing values of all three parameters that resulted in oscillations with intrinsic amplitudes and set-points in the range of 15–30° and 25–75°, respectively, and that spanned the intrinsic frequency range of 5–9 Hz (Fig 6f). We note that during natural whisking behavior, the animal does not necessarily need to keep the intrinsic amplitude and set-point of the vIRt oscillator system within these ranges; however, in order to analyze amplitude modulation due to detuning *per se* in our simulations, we selected this narrow range in order to minimize the potentially confounding effects of amplitude and set-point modulations that are intrinsic to the vIRt-FMN network. The normalized power spectra of the selected oscillations exhibited similar levels of spectral purity, with an average spectral full-width at half maximum value of 2.40 ± 0.36 (mean + std. dev.).

**Fig 7:**
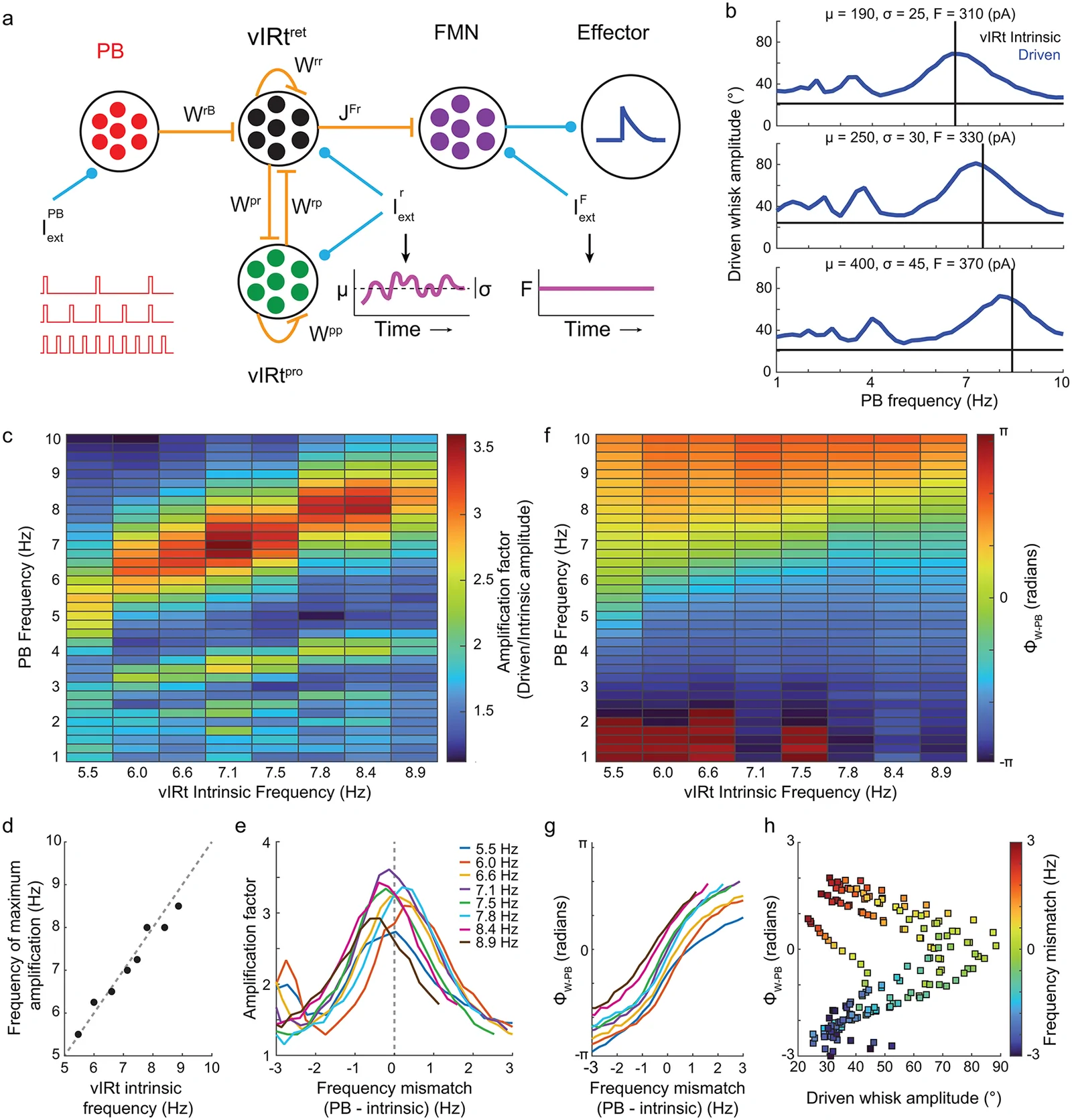
Model whisking CPG network with periodic drive produces oscillations with amplitudes that depend on the alignment of the intrinsic and drive frequencies. (a) Schematic of neural network model of the vibrissa intermediate reticular formation with time-varying external input current to ${\text{vIRt}}^{\text{pro}}$ and ${\text{vIRt}}^{\text{ret}}$ (magenta), constant external input current to FMN (magenta), and PB input to ${\text{vIRt}}^{\text{ret}}$ with $I_{ext}^{PB}\;$as pulse trains with pulse duration 50 ms, amplitude 500 pA over a range of frequencies. Conventions are as in Figs 3a and 6a. (b) Plots of the mean amplitude of the first whisk following each $I_{ext}^{PB}$ pulse at steady state (*driven whisk amplitude*) for 30-second simulations of the network in Fig 7a at $I_{ext}^{PB}$ pulse frequencies ranging from 1-10 Hz. Random variability in each inter-pulse period is 0%. The horizontal and vertical black lines represent the intrinsic amplitude and intrinsic frequency, respectively, of simulations in which $I_{ext}^{PB}$ = 0, as in Fig 6a. Plots of effector output amplitudes using values of *σ*, *µ*, *F* that result in intrinsic vIRt frequencies of 6.6 Hz (top), 7.5 Hz (middle), and 8.4 Hz (bottom) are shown. (c) Heatmap of normalized *driven whisk amplitudes* for simulations of the network in Fig 7a with the *σ*, *µ*, *F* parameter sets in Fig 6f and PB drive frequencies between 1 and 10 Hz. Color corresponds to the *amplification factor*, defined as the *driven whisk amplitude* (blue points in Fig 7b) normalized by the *intrinsic amplitude* of the vIRt network when $I_{ext}^{PB}$ = 0 (black horizontal lines in Fig 7b, see **Materials and Methods**). (d) Plot of the PB input frequency that results in the maximal amplification factor versus the vIRt intrinsic frequency for each of the simulations in Fig 7c. (e) Plots of the amplification factor as a function of the difference between the PB input frequency and the vIRt intrinsic frequency (*frequency mismatch*) for each of the simulations in Fig 7c. (f) Heatmap of the phase of coherence between PB input current pulse onset times and the effector signal, calculated at the PB pulse frequency ($\varphi_{W-PB}$), for simulations of the network in Fig 7a with the *σ*, *µ*, *F* parameter sets in Fig 6f and PB drive frequencies between 1 and 10 Hz. (g) Plots of $\varphi_{W-PB}$ as a function of the frequency mismatch for each of the simulations in Fig 7c, 7f. Conventions are as in Fig 7e (*frequency mismatch*)**, 7f** ($\varphi_{W-PB}$). (h) Plot of $\varphi_{W-PB}$ vs. the *driven whisk amplitude* for the simulations in Fig 7c, 7f for which the frequency mismatch is within the range of ±3 Hz. Color of the data points corresponds to the frequency mismatch. Conventions are as in Fig 7b (*driven whisk amplitude*)**, 7e** (*frequency mismatch*)**, 7f** ($\varphi_{W-PB}$).

The responses of the PB/vIRt/FMN network model to frequency sweeps of PB input current pulse trains (Fig 7a) for different *µ, σ*, and *F* current parameter sets suggest that, qualitatively, the maximum *driven whisk amplitude* is achieved when the PB drive frequency $f_{\text{PB}}$ is close to the vIRt intrinsic frequency $f_{\text{intrinsic}}$, consistent with the theoretical expectation if the network is operating as an entrained driven oscillator system (Fig 7b). To visualize the amplification effects, heatmaps of the *amplification factor* $AF\left(f_{\text{PB}}\right)$ defined by the ratio of the *driven whisk amplitude* $A\left(f_{\text{PB}}\right)$ to the *intrinsic amplitude* $A_{\text{intrinsic}}$ were generated across combinations of drive and intrinsic frequencies. The amplification factor is shown for each of the representative sets of input parameters shown in Fig 6f over the range of PB input frequencies between 1 and 10 Hz (Fig 7c). Across the parameter sets, the PB drive frequency that produces the maximal amplification factor is closely aligned with the vIRt intrinsic frequency (Fig 7d). Finally, an analysis of how the amplification factor varies as a function of the *frequency mismatch* $\Delta f=f_{\text{PB}}-f_{\text{intrinsic}}$ shows a range of whisking amplitudes, with AFs varying approximately linearly between ~1.5 and 3.5 times $A_{\text{intrinsic}}$, can be achieved for Δf within the range of approximately ± 3 Hz (Fig 7e). These simulations indicate that a PB/vIRt/FMN network connected in a manner consistent with anatomical observations [14], [15], [24], [25] could, in principle, use changing input currents to dynamically set the vIRt intrinsic frequency, enabling the animal to modulate its whisking amplitude by detuning from the breathing frequency.

We next evaluated how the modeled whisking-to-PB phase varied as a function of drive frequency and intrinsic frequency, for the representative parameter sets *µ, σ*, and *F* in Fig 6f. Consistent with the observed linear phase shift across drive frequencies for a constant intrinsic frequency (Fig 5b), there was a consistent effector phase shift across parameter sets that depended on Δf (Fig 7f and 7g). For the simulated whisking traces in Fig 7c, 7f with Δf between -3 and 3 Hz, the phase-amplitude curve resembles a sideways “V” shape, with a negative slope for simulations with Δf>0 and a positive slope for simulations with Δf<0 (Fig 7h). Comparing this curve to the behavioral phase-amplitude curve (Figs 2l and 7h): first, there is a substantial offset in the absolute phase values. This phase difference likely reflects the fact that the model analysis uses simulated PB input current pulse times whereas experimental uses measured airflow, which lags pre-inspiratory spiking activity in PB substantially. A rough estimate of the phase lead between PB burst onset and inhalation onset during sniffing is between π and π/4 radians [14] (see **Materials and Methods**), and the observed phase difference between the model predictions and behavioral results appears to be within this range (Figs 2l, 7h). Second, the experimental phase-amplitude curve also exhibits a prominent negative slope for Δf>0, which is consistent with a behavioral strategy in which, during sniffing, the rats adjust the vIRt intrinsic frequency so that it is maintained slightly below the variable sniffing frequency (Δf>0), at least during this behavioral task that encourages large-amplitude exploratory whisking [14,45]. The experimental and model phase-amplitude dependences are thus consistent with the rats using vIRt intrinsic frequency modulation as a strategy for setting whisking amplitude during sniffing.

### Kinematic features of simulated whisking in relation to breathing

**Fig 8:**
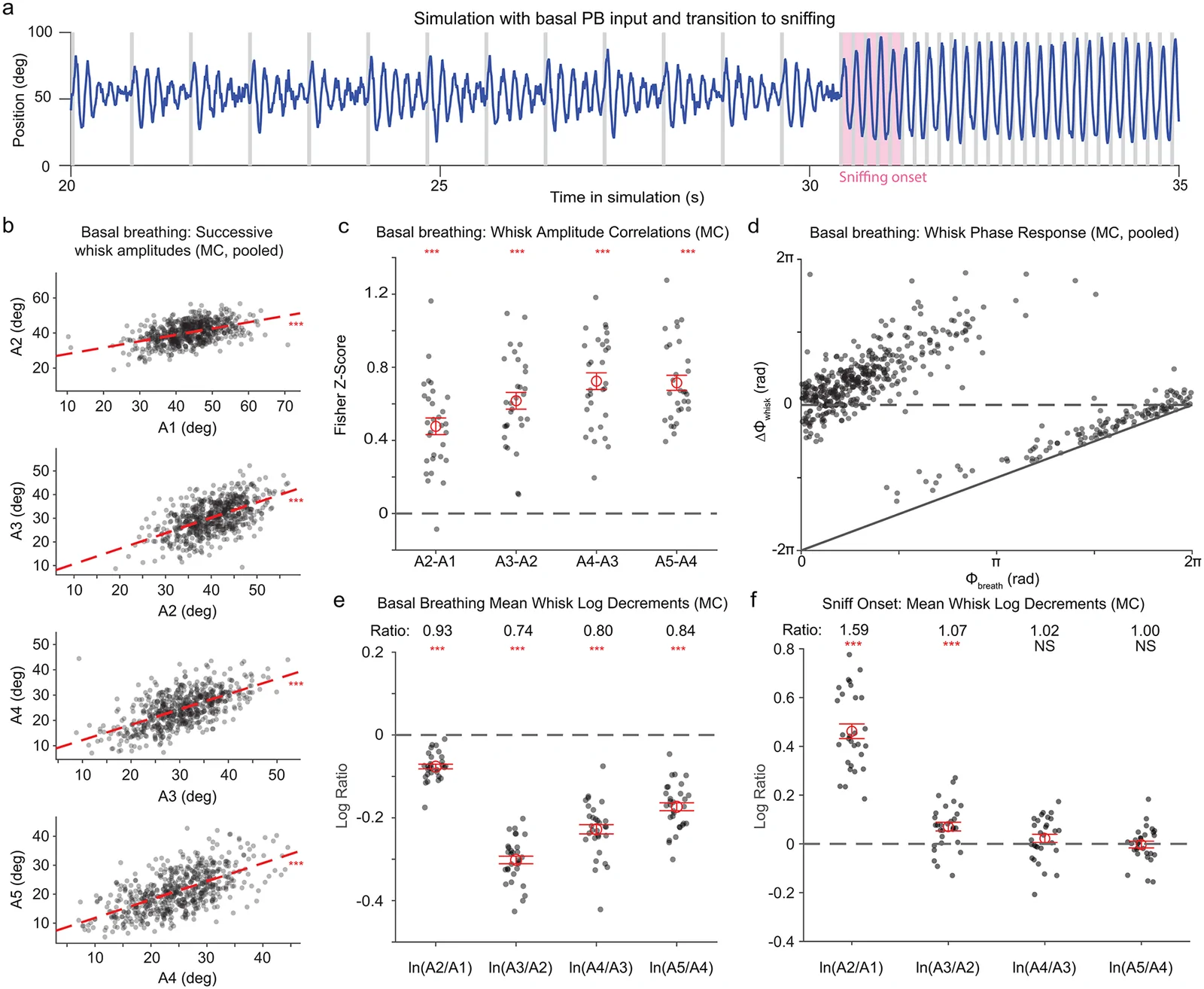
Model whisking CPG network with periodic drive produces oscillations with kinematic features of natural whisking. (a) Sample segment of a simulation of the network in Fig 3a with 30 seconds of $I_{ext}^{PB}\;$as a pulse train with pulse duration 50 ms, amplitude 500 pA, and frequency 1.25 Hz, followed by a transition to 6 Hz. (b) Correlations between $A_{n+\text{1}}$ and $A_{n}$ for all whisks during 1.25 Hz stimulation at steady state (first 15 seconds ignored). Observations are pooled over all breaths in 30 Monte Carlo (MC) simulations with different random instantiations of the connectivity matrices $W^{rB}$, $W^{rr}$, $W^{pp}$, $W^{rp}$, $W^{pr}$, $W^{Fr}$. Conventions are as in Fig 2e. (c) Fisher z-scores $z_{n+\text{1},n}=\text{atanh}\left(r_{n+\text{1},n}\right)$ for correlation coefficients between $A_{n+\text{1}}$ and $A_{n}$ for each of the 30 Monte Carlo simulations. Each gray dot represents the correlation coefficient $\left(r_{n+\text{1},n}\right)\;$for one Monte Carlo simulation. Red circles indicate the mean Fisher z-scores $\overset{―}{z_{n+\text{1},n}}$ and bars represent 95% confidence intervals of the mean. Average correlation coefficients ($\overset{―}{r_{n+\text{1},n}}$) and statistical significance (Student’s *t*-test on $\overset{―}{z_{n+\text{1},n}}\text{≠}\text{0}$): $\overset{―}{r_{\text{2},\text{1}}}=\text{0}.\text{4}\text{4}$; $\overset{―}{r_{\text{3},\text{2}}}=\text{0}.\text{5}\text{5}$; $\overset{―}{r_{\text{4},\text{3}}}=\text{0}.\text{6}\text{2}$; $\overset{―}{r_{\text{5},\text{4}}}=\text{0}.\text{6}\text{1}$.*** p < 0.001 (d) Pooled phase response plot for the 30 Monte Carlo simulations in **panel b** (see **Materials and Methods**). Each gray dot represents 1 pulse in 1.25 Hz $I_{ext}^{PB}$ pulse trains at steady state. (e) Distributions of log ratios $d_{n+\text{1},n}=\;\text{ln}\left(A_{n+\text{1}}/A_{n}\right)$ of successive whisk amplitudes during basal breathing, where A represents the amplitude and n represents the nth whisk after a stimulation at 1.25 Hz. Each gray dot represents the mean log ratio for all the $I_{ext}^{PB}$ pulses at steady state in one simulation. Red circles indicate the mean of the mean log ratios $\overset{―}{\overset{―}{d_{n+\text{1},n}}}$ over the 30 Monte Carlo simulations, and red bars indicate 95% confidence intervals of the mean. Average amplitude ratios $\overset{―}{\overset{―}{A_{n+\text{1}}/A_{n}}}=exp(\overset{―}{\overset{―}{d_{n+\text{1},n}}})\;$are displayed on the top. Statistical significance (Student’s *t*-test on $\overset{―}{\overset{―}{d_{n+\text{1},n}}}\text{≠}\text{0}$): *** p < 0.001 (f) Distributions of log ratios $d_{n+\text{1},n}=\;\text{ln}\left(A_{n+\text{1}}/A_{n}\right)$ of successive whisk amplitudes, where A represents the amplitude and n represents the nth whisk after the transition from 1.25 Hz to 6 Hz $I_{ext}^{PB}$ stimulation in each of the 30 Monte Carlo simulations. Red circles indicate the mean log ratios $\overset{―}{d_{n+\text{1},n}}$ over all simulations, and red bars indicate 95% confidence intervals. Average amplitude ratios $\overset{―}{A_{n+\text{1}}/A_{n}}=exp(\overset{―}{d_{n+\text{1},n}})\;$shown on the top. Statistical significance (Student’s *t*-test on $\overset{―}{d_{n+\text{1},n}}\text{≠}\text{0}$): *** p < 0.001.

Given that the PB/vIRt/FMN network model exhibits entrainment and frequency-dependent amplification when the PB pulse frequency is close to the dominant vIRt intrinsic oscillation frequency, we sought to address whether kinematic signatures of simulated whisking exhibited similar features to those of natural rat whisking (Fig 2). We simulated 30-second PB pulse trains at a basal frequency of 1.25 Hz, followed by a transition to simulated “sniffing” with PB input current pulses at 6 Hz, which is the vIRt intrinsic frequency for this network (Fig 8a). Across 30 Monte Carlo simulations with random instantiations of the connectivity matrices, we observed correlations between amplitudes of successive whisks (Fig 8b), consistent with natural whisking (Fig 2e). Across network connectivity instantiations, correlation coefficients between each successive pair of oscillation cycles were significantly greater than zero for the first 5 cycles following PB stimulation (Fig 8c). An analysis of the phase response of simulated whisking to basal breathing exhibited strong resetting of the rhythm (Fig 8d) as observed in previous experimental observations and associated simulations [14,24], consistent with the model parameters for PB drive to vIRt being strong enough to influence the ongoing intrinsic vIRt rhythm. Additionally, simulated whisk amplitudes decreased successively across intervening oscillation cycles (Fig 8e) as in natural whisking (Fig 2d). A supplementary analysis using an artificially low PB stimulation rate (0.5 Hz pulses) to produce more intervening whisk cycles per pulse demonstrated that successive whisk amplitudes converge at a steady-state value of approximately 19° after 8 whisk cycles, close to the *intrinsic amplitude* of 18° for this network (Figs 5c, S3). Finally, we observed significant increases in successive whisk amplitudes over the first 3 oscillation cycles following the transition to sniffing (Fig 8a, 8f), which is also observed in natural whisking (Fig 2g, 2h). All in all, there is broad alignment in kinematic signatures between the PB/vIRt/FMN network model that exhibits entrainment and frequency-selective amplification and observations of natural rat exploratory (i.e., sniffing locked) whisking, consistent with the hypothesis that there is entrainment and frequency-dependent amplification between whisking and sniffing.

## Discussion

In this study, we have investigated how nervous systems can control the amplitude of rhythmic movements via interacting neuronal oscillator circuits, using rodent whisking as a model system. A re-analysis of synchronous whisking and breathing measurements in rats (Fig 1) shows that during basal breathing the whisking frequency distribution is broad and peaks slightly above the typical sniffing frequency (Fig 2a). This observation suggests that under basal breathing conditions the whisking oscillator intrinsic frequency is slightly offset from the typical sniffing frequency, although whisking and breathing movements occur in a 1:1 phase-locked manner during sniffing. Additionally, it has previously been observed that during slow breathing the intrinsic whisking oscillator frequencies on the left versus right sides of the face are themselves even sometimes mismatched [25]. These observations are consistent with a variable vIRt intrinsic frequency that rodents could modulate to “tune in” or “detune” from breathing to modulate whisking amplitude. Correspondingly, whisking kinematics exhibit signatures of being generated by a periodically driven oscillator system (Figs 1, 2). These observations indicate that the whisking neuronal oscillator is likely to be entrained by breathing during periods of synchronous 1:1 whisking and sniffing [22]. Further, an analysis of the phase of whisking during sniffing suggests that rats actively modulate the whisking oscillator intrinsic frequency during sniffing and that phase is correlated with whisking amplitude, also consistent with the detuning hypothesis (Fig 2i-2l).

To assess whether the known and inferred connectivity of the whisking oscillator circuit can support entrainment with breathing, we constructed an adaptive exponential integrate-and-fire neuronal network model of the whisking oscillator circuit and its drive from a breathing oscillator circuit based on a simplification of a previous conductance-based neuronal network model [24] (Fig 3). The present model predicts that neuronal adaptation would endow the network with both rhythmogenesis and the persistence of perturbation effects from previous synaptic inputs over several oscillation cycles. If subsequent stimulation arrives during the constructive phase of the oscillation cycle, constructive interference leads to an enhancement in the amplitude of the subsequent cycle, causing the whisking amplitude to increase. Indeed, simulations of rhythmic inputs to vIRt from PB at different drive frequencies show that whisking amplitude is maximized when the vIRt intrinsic frequency matches the drive frequency (Figs 4, 5). As in the previous Golomb et al. model [24], inter-pool inhibition (${\text{vIRt}}^{\text{pro}}$ to ${\text{vIRt}}^{\text{ret}}$ and vice versa) that is slightly stronger than intra-pool inhibition produces anti-phasic activation in the two pools while also maintaining high variability in spike timing, with CV2 values close to 1 across a range of PB drive frequencies, as observed *in vivo* [15,24]. Our model also predicts that for a *constant* vIRt intrinsic frequency, the phase of whisking should shift with PB drive frequency in the 1:1 regime (Fig 5b). The behavioral observation that whisking-to-sniffing phase is not significantly correlated with sniffing frequency but is correlated with amplitude is consistent with the hypothesis that rats actively co-modulate PB and vIRt frequencies (Fig 2i-2l); however, the quantitative extent of this co-modulation is uncertain due to variability in the phase relationships between burst timing of individual PB neurons and inhalation onset during sniffing [14] (see **Materials and Methods**).

Because whisking amplitude and frequency appear to be task dependent and therefore under volitional control [9,45] on the time scale of 1 second [18], we sought to identify whether the intrinsic vIRt frequency could be changed by descending neuronal inputs, rather than changes in network connectivity. Additionally, since ${\text{vIRt}}^{\text{pro}}$ and ${\text{vIRt}}^{\text{ret}}$ neurons are observed to be intermingled, we made the parsimonious assumption that descending drive to ${\text{vIRt}}^{\text{pro}}$ and ${\text{vIRt}}^{\text{ret}}$ populations is symmetric, though in principle these populations could be targeted differentially by higher order brain regions. Our simulations demonstrated that in order to change the vIRt intrinsic frequency independently of the intrinsic amplitude and set-point, both the mean and the amplitude of noise fluctuations in the input current would have to be increased together (Fig 6). These observations demonstrate that the introduction of noisy input currents to vIRt is sufficient to produce changes in the vIRt intrinsic frequency that modulate amplitude by biasing the vIRt network intrinsic frequency towards or away from the sniffing frequency (Fig 7). The model further suggests that whisking-to-sniffing phase decreases with increased whisking amplitude, as observed behaviorally (Figs 2l, 7h), for vIRt intrinsic frequencies that are biased to be below the sniffing frequency.

Importantly, the model outputs exhibit similar kinematic signatures of entrainment and frequency-selective amplification to natural rat whisking (Figs 2, 7–8), and this model explains the curious observation of intervening whisks during basal breathing [14] as side effects of a driven oscillator circuit for which the effects of the drive signal persist and decay over multiple oscillation cycles. Further, the alignment between the model output (Fig 8) and behavioral observations (Fig 2) makes the prediction that intrinsic electrophysiological properties of ${\text{vIRt}}^{\text{pro}}$ and ${\text{vIRt}}^{\text{ret}}$ neurons should support frequency-selective amplitude modulation, perhaps due to the time scale of neuronal adaptation. There exist numerous potential biophysical mechanisms that support adaptation on relevant time scales [46].

**Fig 9:**
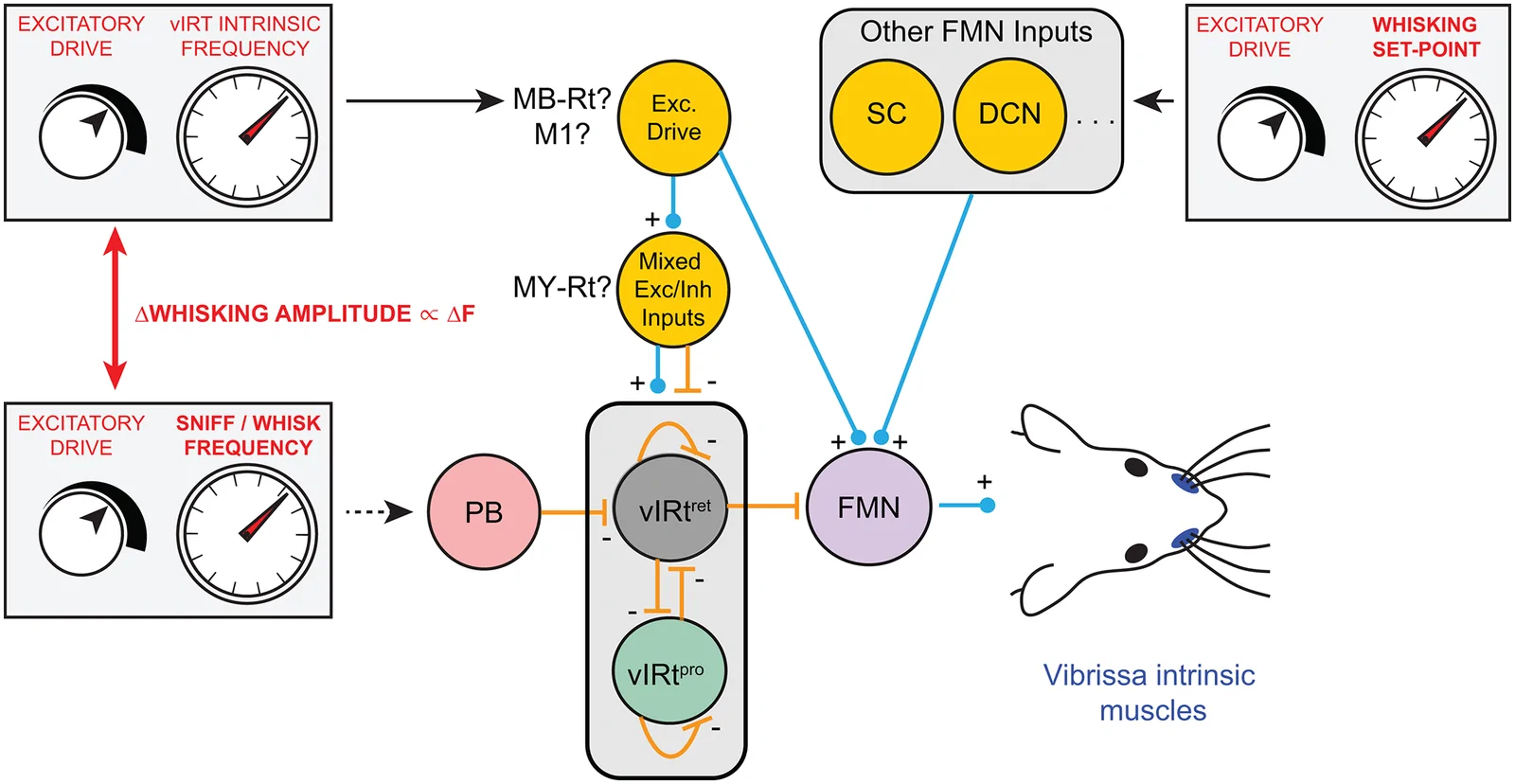
A proposed anatomical neuronal circuit that controls whisking amplitude by frequency detuning. In the proposed circuit, ${\text{vIRt}}^{\text{pro}}$ and ${\text{vIRt}}^{\text{ret}}$ neuron populations are driven symmetrically by an upstream region that provides mixed excitatory and inhibitory presynaptic inputs. Based on previous studies, this input could come from the medullary reticular formation (MY-Rt), either by other neurons in the IRt, or by neurons in the Gi [15]. This region is itself driven by an upstream region that provides excitatory presynaptic inputs to the MY-Rt, such as the midbrain reticular formation (MB-Rt) or primary motor cortex (M1) [54], which sets the vIRt intrinsic frequency. This region(s) may also provide inputs to facial motor neurons (FMN) that protract the vibrissae. In this hypothetical anatomical circuit, the whisking frequency is set by excitatory or modulatory inputs to the preBӧtzinger complex (PB) that set breathing frequency. Whisking set-point is set by excitatory inputs to FMN including the MB-Rt, M1, deep cerebellar nuclei (DCN) and superior colliculus (SC). Whisking amplitude is controlled by the mismatch between the vIRt intrinsic frequency and the breathing frequency.

Our model further predicts that the intrinsic vIRt frequency can be modulated by controlling the statistics of “noisy” input currents to the vIRt network. Perhaps one of the most biologically plausible mechanisms to introduce these types of noise fluctuations into a neuronal oscillator circuit is mixed excitation and inhibition, ensuring that the circuit can oscillate over a wide dynamic range [47,48]. From which neuroanatomical regions might such a source of mixed excitatory and inhibitory inputs arise? While in principle the sources of excitation and inhibition could come from different anatomical locations, a more parsimonious solution would be if they come from the same brain area, enabling them to be easily co-modulated. Recently, Takatoh et al. performed dG-rabies tracing to map presynaptic inputs to vIRt PV+ neurons, a cell type that is essential for whisking [15]. Most long-range descending inputs to vIRt PV+ neurons are exclusively excitatory; however, inputs from the gigantocellular reticular formation (Gi), as well as other neurons throughout the intermediate reticular formation (IRt), provide mixed excitation and inhibition. However, since rabies-based input mapping is likely not exhaustive [49], other sources of mixed excitation and inhibition input are possible. Nonetheless, based on these observations, we propose that the medullary reticular formation (MY-Rt), which includes both Gi and IRt, is a likely source for this mixed descending drive (Fig 9). The possibility that the MY-Rt provides descending drive to a CPG that transforms tonic spiking into rhythmic output aligns with its role in other motor systems. For example, in anesthetized rodent preparations in which chewing is induced by tonic stimulation of the cortical masticatory area, cortically evoked tonic and rhythmic activation of different populations of MY-Rt neurons was observed [50]. Similarly, in the locomotor system, stimulation of the mesencephalic locomotor region (MLR) in the midbrain reticular formation (MB-Rt) produces tonic activation of reticulospinal neurons in the MY-Rt, which activates oscillator circuits in the spinal cord [51]. As is the case with MY-Rt neurons presynaptic to vIRt, the spinal-projecting MY-Rt population is thought to be comprised of a mixture of excitatory and inhibitory neurons [52,53].

Our model also suggests that when increasing the vIRt frequency by applying external input currents to vIRt neurons, parallel excitatory drive to FMNs is also required to avoid tonic suppression of motor outputs with increasing levels of inhibition from vIRt (Fig 6). Because descending excitatory inputs from primary motor cortex (M1) [50] and MB-Rt [51] can activate chewing and locomotor CPGs, respectively, via projections to the MY-Rt, and because these areas also innervate vibrissa facial motor neurons [54], we hypothesize that these are likely candidate areas to modulate the vIRt intrinsic frequency via parallel projections to vibrissa FMNs and MY-Rt neurons upstream of the vIRt (Fig 9). Additionally, other prominent inputs to FMNs, such as from the deep cerebellar nuclei (DCN) and superior colliculus (SC), are candidate sites (Fig 9) to influence vibrissa set-point under appropriate behavioral circumstances like orienting [10], turning, and running [55]. Therefore, based on our findings together with these anatomical observations, we propose that the following mechanisms enable rats to control the frequency, amplitude, and set-point of exploratory whisking relatively independently: (1) frequency modulation directly by modulation of the sniffing frequency in the breathing oscillator circuit; (2) amplitude modulation by descending excitatory drive to a mixed excitatory/inhibitory population in the MY-Rt that detunes the vIRt intrinsic rhythm from breathing; and (3) set-point modulation by excitatory input to facial motor neurons (Fig 9). This separation of frequency, amplitude, and set-point [18] would enable the full range of flexible whisking behaviors observed in rodents.

Our findings in this study support the more general concept that differences in frequency among interacting neuronal networks are not noise, but instead are ways to control neuronal synchrony. Recent evidence suggests that oscillations in classical brain frequency bands such as gamma and theta often differ in frequency by several Hz in different or even the same brain regions. Such detuning (slight difference in frequency) between weakly coupled oscillators allows the brain to limit synchrony (prevent seizures), create travelling waves, and regulate phase-locking thus effective connectivity, all reviewed in [23]. The frequency matching between hierarchically driven oscillators has also been shown to be important for speech perception [56], and varying the frequency of oscillatory input to neuronal network models of working memory can gate transitions between functional states [57,58]. Furthermore, the resonance effect of tuning external inputs with intrinsic subthreshold voltage oscillations has been demonstrated to be important for spatial mapping in hippocampal grid cells [59]. Here we extend these concepts to the domain of motor control and show that frequency detuning and entrainment between two network-level oscillatory circuits can also lead to analog control of rhythmic movement amplitudes. We demonstrate that basic patterned movement sequences such as whisking, which is controlled by interacting CPGs, can exhibit amplitude modulation based on their relative frequencies. We show that detuning represents a computationally efficient mechanism that endows CPG circuits with the flexibility to produce varied motor patterns while still respecting the biomechanical constraints of the motor plant.

Finally, we note that the vibrissa motor plant is more complex than the present model, which considers only the activation of the intrinsic musculature. In reality, the vibrissae are embedded in the complex tissue of the mystacial pad, with multiple muscle groups and its own viscoelastic properties. Though the pad itself is thought to be heavily damped, modulating pad stiffness via blood flow as well as extrinsic muscle tension could provide additional nodes for controlling vibrissa kinematics beyond the vIRt [16,60]. Determining how such modulation of the motor plant itself may interact with the PB/vIRt/FMN circuit to influence vibrissa movement and active sensory perception for embodied cognition [61] will require experimental and modeling studies that unite neural and biomechanical levels of analysis.

## Materials and methods

### Neuron model

The neuronal network was simulated using the Adaptive Exponential Integrate-and-Fire (AdEx) model [44]. The membrane potential V(t) and adaptation current w(t) for each neuron were governed by the following differential equations numerically implemented using the Euler method:


$$\begin{array}{c} \begin{array}{c} C_{m}\frac{dV}{dt}=-g_{L}\left(V(t)-E_{L}\right)+g_{L}\Delta V_{Exp}(t)-w(t)+I_{syn}(t)+I_{ext}(t) \end{array} \end{array}$$ (1)

$$\begin{array}{c} \begin{array}{c} {\Delta V_{Exp}(t)\;=\Delta }_{T}\text{exp}\left(\frac{V(t)-V_{T}}{\Delta_{T}}\right) \end{array} \end{array}$$ (2)

$$\begin{array}{c} \frac{dw}{dt}=\frac{a\left(V(t)-E_{L}\right)-w}{\tau_{w}}+b\sum_{k}\delta \left(t-t_{k}\right) \end{array}$$ (3)

where ${\text{C}}_{\text{m}}$ is the membrane capacitance, $g_{L}$ is the leak conductance, $E_{L}$ is the leak reversal potential, $\Delta V_{Exp}(t)$ is the exponential term (a simplification of the Hodgkin-Huxley type sodium current), $\Delta_{T}$ is the slope factor (sharpness of action potential initiation), $V_{T}$ is the effective spike initiation threshold, $I_{syn}(t)$ is the synaptic current, $I_{ext}(t)$ is the external current, a is the subthreshold adaptive conductance, $\tau_{w}$ is the subthreshold adaptive current time constant, b is the spike-triggered adaptative current, and δ() denotes the Dirac delta function. That is, at every time point $t_{k}$ when V(t) exceeded a spike detection threshold $V_{spike}$ (0 mV), a spike was recorded, the membrane potential was reset to a reset potential $V_{reset}$, and the adaptation current was incremented by b (w→w+b). The neuron remained clamped at $V_{reset}$ for an absolute refractory period $T_{ref}$ (for simplicity, we have set $T_{ref}=\text{0}$; inter-spike intervals were never < 1 ms in our simulations).

Two distinct parameter sets were used to model different firing phenotypes and were adapted from [39]. “Non-adaptive” neurons (used for the preBötzinger complex and facial motor nucleus populations) were modeled with zero adaptation (a=0nS, b=0pA). “Adaptive” neurons (used for the vIRt-retraction and vIRt-protraction populations) utilized both subthreshold and spike-triggered adaptation to replicate bursting dynamics. The complete sets of parameter values are given below:

**Table:** 

	${𝐂}_{𝐦}$ (pF)	$g_{L}$ (nS)	$E_{L}$ (mV)	$\Delta_{T}$ (mV)	$V_{T}$ (mV)	𝐚 (nS)	$\tau_{w}$ (ms)	b (pA)	${𝐕}_{spike}$ (mV)	${𝐕}_{reset}$ (mV)	${𝐓}_{ref}$ (ms)
Non-adaptive	100	12	-70	2	-50	0	N/A	0	0	-58	0
Adaptive	100	12	-70	2	-50	-11	130	30	0	-48	0

For simplicity, we do not model variability in the intrinsic neuron properties for either the “adaptive” or “non-adaptive” pools.

### Network architecture and connectivity

The model network consisted of four interconnected populations: the preBötzinger complex inhibitory inhalation neurons (PB, N=200), the vIRt-retraction inhibitory population (${\text{vIRt}}^{\text{ret}}$, N=800), the vIRt-protraction inhibitory population (${\text{vIRt}}^{\text{pro}}$, N=400), and the vibrissa motor neurons in the facial motor nucleus (FMN, N=100). These rough estimates were derived from past experimental studies and rounded to the nearest hundred. Specifically:

The number of PB neurons were based on an estimate of a total of 1000 PB neurons per side [34], approximately 80% of which are inhalation-inducing at postnatal day 6–9, a time point at which respiratory networks are thought to stabilize [35]. Of inhalation-inducing neurons, 30% are thought to be inhibitory [36], yielding ~200 neurons.

The number of neurons in vIRt was estimated based on the approximate size of the region of the cluster of facial premotor neurons in the peri-ambiguus region from published data [14]. This region is estimated to be roughly 1000um rostrocaudally, 500um dorsoventrally, and 300um mediolaterally. In comparison, the PB is estimated to be 400um rostrocaudally, 300um dorsoventrally, and 300um mediolaterally based on published extracellular recordings [62]. The ratio of these respective areas is approximately 4:1. Assuming 1000 PB neurons and equal neuronal densities between PB and vIRt, which are both in the intermediate zone of the medullary reticular formation, the number of neurons in the anatomical area of the vIRt in the rat is estimated at ~4000 per side.

To estimate the fraction of excitatory versus inhibitory neurons in vIRt, independently of whether or not they project to FMNs [14,54], we counted neurons in the Allen Brain Atlas mouse in situ hybridization database [37] in a 500um x 500um region encompassing the vIRt in a single section immediately rostral to the lateral reticular nucleus. We counted 77 *VGAT* neurons (60%) and 56 *VGLUT2* neurons (40%). We made a default assumption that 50% of vIRt neurons are whisking-related, yielding 1200 inhibitory, whisking-related vIRt neurons. Of these, approximately 66%, or 800, are ${\text{vIRt}}^{\text{ret}}$ [14,25] and 33%, or 400 are ${\text{vIRt}}^{\text{pro}}$. We used 100 facial motor neurons based on previous estimates [38].

Connectivity was defined with the assumption of sparse, random projections. For each projection pair (S→T), a connectivity matrix $W^{TS}$ of size $N_{target}\times N_{source}$ was generated. A connection from neuron j to neuron i was established (nonzero weight) with probability $P_{conn}$ (Bernoulli trial). If established, the synaptic weight was set to a value of $I_{syn}$. Synaptic currents $I_{syn}$ decayed exponentially with a time constant $\tau_{syn}=\text{1}\text{0}$ ms, which approximates the behavior of inhibitory GABA_A_ and glycine receptors [63,64]. For all main figures, the connection probability $P_{conn}$ and peak synaptic currents $I_{syn}$ were set as follows: $\text{PB}\;\to \;{\text{vIRt}}^{\text{ret}}$($P_{conn}=\text{0}.\text{4}$, $I_{syn}=-\text{0}.\text{0}\text{4}\;pA$), ${\text{vIRt}}^{\text{ret}}↔{\text{vIRt}}^{\text{pro}}$ (inter-pool mutual inhibition, $P_{conn}=\text{0}.\text{1},\;I_{syn}=-\text{3}\;pA$), and ${\text{vIRt}}^{\text{ret}}\to FMN$ ($P_{conn}=\text{0}.\text{1},\;I_{syn}=-\text{3}\;pA$). Intrapopulation (intra-pool) connectivity was included for ${\text{vIRt}}^{\text{ret}}$ and ${\text{vIRt}}^{\text{pro}}$ populations ($P_{conn}=\text{0}.\text{1},\;I_{syn}=-\text{2}\;pA$). In S1 Fig, ${\text{vIRt}}^{\text{ret}}↔{\text{vIRt}}^{\text{pro}}$ inter-pool mutual inhibition and ${\text{vIRt}}^{\text{ret}}$, ${\text{vIRt}}^{\text{pro}}$ intra-pool inhibition peak synaptic currents ($\;I_{syn}$) were varied between 0 and −8 pA in steps of 0.5 pA with all other parameters set as above. In S2 Fig, $\text{PB}\;\to \;{\text{vIRt}}^{\text{ret}}$ inhibition was varied between 0 and −0.16 pA in steps of 0.04 pA with all other parameters set as above.

### Effector model

The simulated whisker position, or “effector” (E), was driven by the summed spiking activity of the vibrissa motor neurons in the facial motor nucleus (FMN). The effector dynamics were modeled using an alpha function response to each spike:


$$\begin{array}{c} \frac{d^{\text{2}}E}{dt^{\text{2}}}+\frac{\text{2}}{\tau_{\alpha }}\frac{dE}{dt}+\frac{\text{1}}{\tau_{\alpha }^{\text{2}}}E(t)=\frac{X_{scale}}{\tau_{\alpha }^{\text{2}}}\sum_{k}\delta \left(t-t_{k}\right) \end{array}$$ (4)

where $\tau_{\alpha }=\text{1}\text{0}$ ms is the time constant governing both the rise and decay phases of the movement and $X_{scale}=2{}^{\circ}$ determines the angular displacement per spike. The effector model is based on the measured transfer function between single spikes and vibrissa assuming linear summation of motor neuron spikes [38]. This model was implemented numerically using the Euler method as a system of two first-order differential equations via an intermediate variable X:


$$\begin{array}{c} \begin{array}{c} X(t)\leftarrow X(t)+X_{scale}\cdot S_{FMN}(t) \end{array} \end{array}$$ (5)

$$\begin{array}{c} \begin{array}{c} X(t+\Delta t)=X(t)+\left(-\frac{X(t)}{\tau_{\alpha }}\right)\Delta t \end{array} \end{array}$$ (6)

$$\begin{array}{c} \begin{array}{c} \text{E}(\text{t}+\Delta \text{t})=\text{E}(\text{t})+\left(\frac{\text{X}(\text{t})-\text{E}(\text{t})}{\tau_{\alpha }}\right)\Delta \text{t} \end{array} \end{array}$$ (7)

where $S_{FMN}(t)\;$represents the total count of spikes generated by the FMN population at time t. For simplicity, we assumed a 0-ms delay between FMN spikes and the initiation of movement.

### External inputs and simulation protocol

All neurons received a background external excitatory input current $I_{ext}$ consisting of population-dependent static mean component $I_{mean}\;$and time-varying fluctuation $I_{noise}$.


$$\begin{array}{c} \begin{array}{c} I_{ext}(t)=I_{mean,pop}+I_{noise,pop}(t) \end{array} \end{array}$$ (8)

The time-varying fluctuation $I_{noise}\;$was modeled as an Ornstein-Uhlenbeck process, described by the stochastic differential equation:


$$\begin{array}{c} \begin{array}{c} dI_{noise}(t)=-\frac{\text{1}}{\tau_{noise}}I_{noise}(t)dt+\sigma_{noise}\sqrt{\frac{\text{2}}{\tau_{noise}}}dW_{t} \end{array} \end{array}$$ (9)

where $\tau_{noise}$ is the correlation time constant, $\sigma_{noise}$ is the asymptotic standard deviation of the noise, and $W_{t}$ is a Wiener process. Such a process is low-pass filtered Gaussian white noise and is well-established for modelling synaptic noise in literature [42,43]. Numerically, this was implemented using the exact discrete-time update formula to ensure correct variance for any time step Δt:


$$\begin{array}{c} \begin{array}{c} I_{noise}(t+\Delta t)=I_{noise}(t)\text{exp}\left(-\frac{\Delta t}{\tau_{noise}}\right)+\sigma_{noise}\sqrt{\text{1}-\text{exp}\left(-\frac{\text{2}\Delta t}{\tau_{noise}}\right)}N(\text{0},\text{1}) \end{array} \end{array}$$ (10)

where N(0,1) represents a random number drawn from a standard normal distribution at each time step.

For the purposes of maintaining the excitatory-inhibitory balance as $\mu_{vIRt}$ and $\mu_{FMN}$ were varied (Fig 6a), it was sufficient to introduce variability in $\sigma_{noise,\;vIRt}$ (default 0 pA) and set $\tau_{noise,vIRt}=\text{5}\;ms$, $\sigma_{noise,\;PB}=\text{0}\;pA$, $\sigma_{noise,\;FMN}=\text{0}\;pA$, and $\sigma_{\text{pop}}=\text{0}\;pA$ across all populations. Default values of mean external input currents were given by $\mu_{PB}=\text{0}\;pA$, $\mu_{vIRt}=\text{1}\text{2}\text{0}\;pA$ (same for ${\text{vIRt}}^{\text{ret}}$ and ${\text{vIRt}}^{\text{pro}}$), $\mu_{FMN}=\text{2}\text{8}\text{0}\;pA$ for the simulations in Figs 3–5. For simplicity in the results section and figures, we defined $\mu :=\mu_{vIRt}$(varied from -20–520 pA, in 30 pA steps in Fig 6c), $\sigma :=\sigma_{noise,\;vIRt}$ (varied from 0 to 60 pA, in 5 pA steps in Fig 6c), and $F:=\mu_{FMN}$ (varied from 270 to 430 pA, in 20 pA steps in Fig 6c) as the parameters to be altered to vary intrinsic vIRt oscillation frequencies in Fig 6 and Fig 7. To simulate respiratory drive, the PB population received an additional external excitatory input current pulse train (added to $I_{ext,PB}$) [24] with inter-pulse period $\text{Perio}{\text{d}}_{\text{1}}$ which was varied between 0.1 and 1s, or 1 and 10 Hz.

For simulations which modeled random variability in the breathing rate (Fig 5a, 5b), external excitatory input current pulse trains were constructed to allow for inter-cycle duration variability. Specifically, pulse onset times were generated sequentially with a cycle duration taken from a uniform distribution with mean $\text{Perio}{\text{d}}_{\text{1}}$ and range ${PerVar}_{\text{1}}$. The range was allowed to vary according to a fixed ratio $PerVarPerc_{\text{1}}=(PerVar_{\text{1}}/\text{Perio}{\text{d}}_{\text{1}})\cdot \text{1}\text{0}\text{0}\%$.

For simulations that modeled the transition period between basal respiration and sniffing (Fig 8), the total simulation duration ($T_{max}$) was divided into two epochs separated by a transition time $t_{trans},$ defined by a user-specified ratio $R_{trans}$ (default 0.75):


$$\begin{array}{c} \begin{array}{c} t_{trans}\;=\;T_{max}\;\cdot \;R_{trans} \end{array} \end{array}$$ (11)

*Epoch 1 (Basal Respiration)*: From t=0 to ${\text{t}}_{\text{trans}}$, excitatory input current pulse trains had an inter-pulse period of $\text{Perio}{\text{d}}_{\text{1}}$ (800 ms or 1.25 Hz) and range ${PerVar}_{\text{1}}$ (default 0 ms).

*Epoch 2 (Sniffing)*: From ${\text{t}}_{\text{trans}}$ to ${\text{T}}_{\text{max}}$, the stimulation switched to generating cycle durations with $\text{Perio}{\text{d}}_{\text{2}}$ (166 ms or 6 Hz) and range ${PerVar}_{\text{2}}$ (default 0 ms). To prevent discontinuities or overlapping pulses at the transition boundary, the first pulse of the second epoch was calculated relative to the last pulse and cycle duration of the first epoch.

For simulations which modeled the return to steady state whisk amplitude following perturbation by excitatory input current pulses (S3 Fig), an artificially low, non-physiological simulated basal respiration period was used (2000 ms or 0.5 Hz) to allow relaxation to steady state, and amplitudes of the first 10 whisks following PB pulses were analyzed.

For all simulations in which input from PB was applied, the pulse width was fixed at $W_{pulse}=\text{5}\text{0}\;ms$ and the pulse amplitude was fixed at $A_{pulse}=\text{5}\text{0}\text{0}\;\text{pA}$.

### Simulation length and temporal resolution

All simulations were performed with a time step Δt = 0.1 ms. Total simulation duration was $T_{max}=$ 30,000 ms (30 s) for single-epoch simulations and 40 s for dual-epoch simulations with a PB frequency transition.

### Network entrainment and detuning protocol

To investigate the interaction between the vIRt/FMN network’s intrinsic frequency and the external respiratory drive, we utilized a two-stage simulation protocol.

- *Stage 1: Intrinsic Parameter Characterization.* “Intrinsic” simulations were conducted across the parameter space defined above (that is, where μ, σ, and F are varied systematically) to determine the natural oscillatory characteristics (intrinsic frequency, intrinsic amplitude, and set-point) of the vIRt network in the absence of respiratory drive. Baseline oscillatory regimes were identified based on a set of stability criteria: steady-state amplitude between 15° and 30°, and a set-point between 25° and 75°, denoted as the “target range”. From this map, specific parameter sets were selected by inspection to represent distinct “intrinsic phenotypes” with dominant frequencies spanning approximately 5.5 Hz to 9.0 Hz.
- *Stage 2: Driven Detuning Simulations.* For each selected intrinsic phenotype, we performed “Driven” simulations to assess detuning. The network parameters were re-initialized to the selected values, and the respiratory drive frequency ($f_{d}$) was varied from 1.0 Hz to 10.0 Hz in 0.25 Hz increments. $PerVarPerc_{\text{1}}$ was set to 0%.

### Randomization and monte carlo simulations

Random number generation was required for the construction of the connectivity matrix $W^{TS}$ (all simulations), the creation of noisy input currents (Figs 6, 7), and the creation of variable periods (Fig 5a). In most simulations, we used the Mersenne Twister as the default algorithm and 1 as the default seed.

To assess the robustness of the network dynamics against stochastic variability, 30 randomized replicates were performed for each parameter combination. To ensure independence across different repetitions in these Monte Carlo simulations, we used the Threefry 4x64 generator with 20 rounds as the random number generation algorithm. For each repetition, the random number generator was re-initialized with a unique seed (saved for each simulation for reproducibility) that in turn is generated randomly by drawing integers from a uniform distribution on the interval $\left[\text{1},{\text{2}}^{\text{3}\text{2}}-\text{1}\right]$. For the purposes of this paper (Figs 5c, 8, S3), this randomization introduced variability at the structural level, as the sparse connectivity matrices $W^{TS}$ were regenerated for each simulation, creating unique random topologies while maintaining the fixed connection probabilities ($P_{conn}$). This approach ensured that the observed phasic and rhythmic behaviors were emergent properties of the network architecture rather than artifacts of a specific wiring realization.

### Data analysis

#### Experimental data source.

Publicly available raw breathing and whisking data [14,24] were re-analyzed with data collection detailed previously [14]. Briefly, head-fixed Long-Evans rats were implanted with a thermocouple in the nasal cavity, and the vibrissa angle was monitored and tracked with a Basler A602f camera. Simultaneous breathing and whisking data were collected in 10-second epochs. Across 7 animals and 25 sessions, there were 467 epochs analyzed.

#### Respiratory signal processing.

For rat breathing data, the raw thermocouple voltage signal was band-pass filtered between 1 and 15 Hz with a 3-pole Butterworth filter in both forwards and backwards directions. Respiratory peaks (inhalations) were identified on the filtered signal with the MATLAB findpeaks() function using the following criteria [1] minimum prominence of 15% of the total unfiltered signal range and [2] minimum inter-peak distance of 30 ms.

#### Whisk detection and feature extraction.

Whisk peaks (retraction onset) and valleys (protraction onset) were detected from the whisker angle (experiment) or the effector position trace (simulation). For simulated data, analysis was restricted to the latter portion of each simulation (the first 15 seconds were ignored) to avoid initial transient dynamics. The raw signal was first band-pass filtered between 3 and 25 Hz with a 3-pole Butterworth filter in both forwards and backwards directions to isolate whisking frequencies. Peak and valley times were identified on the filtered signal with the MATLAB findpeaks() function using the following criteria: (1) minimum prominence of 5% of the total unfiltered signal range and at least 5°. (2) minimum inter-peak distance of 30 ms, and (3) maximum whisk duration (inter-valley interval) of 250 ms. These detection parameters were comparable to prior analyses of the same dataset [14] and were validated by inspection of the detected peaks and valleys. The amplitude of each whisk was defined as the difference between the peak value and the average of the preceding and succeeding valley values on the raw signal (Fig 2b, 2f). If no preceding or succeeding valley exists, the peak amplitude is considered invalid.

#### Spectral analysis for experimental data.

To facilitate classification of breathing behavior into sniffing versus basal breathing, experimental data were first divided into non-overlapping 2-second segments. To classify the behavioral state of each segment, instantaneous metrics were calculated using the Hilbert transform on the band-pass filtered signals (same filter parameters as above). For each segment, the *mean whisk amplitude* was calculated as twice the mean of the amplitude of the Hilbert transform envelope. Segments were included in the analysis only if the mean whisk amplitude exceeded a minimum threshold (10°). These whisking segments were further categorized by the mean instantaneous breathing frequency (first derivative of the phase): “Sniffing” segments were defined by a mean instantaneous breathing frequency >5 Hz, “Basal breathing” segments were defined by a mean breathing frequency <3 Hz. The *mean sniff frequency* is defined as the mean instantaneous breathing frequency for segments classified as “sniffing”.

Power spectra were computed for the unfiltered but mean-subtracted continuous whisking and breathing segments using the multi-taper method with discrete prolate spheroidal sequences (Chronux toolbox function mtspectrumc [65]). For trial-averaged calculations of power spectra for experimental segments (2 seconds duration), to reach a frequency resolution of 0.5 Hz, we utilized a time-half-bandwidth product of 1 and a single taper. Power spectra were normalized such that the integral of the power spectral density over the frequency band of interest (0–15 Hz) equaled 1. For single segment calculations of the coherence between whisking and sniffing, the continuous whisking signal and the point process of sniff onset times were used. Spectral coherence was computed using the multi-taper method for point to continuous processes (Chronux toolbox function coherencycpt), which estimates the cross-spectrum normalized by the individual power spectra. Coherence phase was defined as protraction onset relative to inhalation onset, so that negative values indicate a whisking phase lead. To reach a frequency resolution of 1.5 Hz in single segments, we utilized a time-half-bandwidth product of 3 and 5 tapers. Coherence magnitude and phase values at the mean sniff frequency (|$C_{WS}$| and $\varphi_{WS}$, respectively) were calculated by linear interpolation of the coherence magnitude vs. frequency and coherence phase vs. frequency curves. The statistical significance of the single segment coherence values was computed via the Jackknife method.

#### Spectral analysis for model simulations.

Simulated effector data were analyzed during the steady-state period (last 15 seconds of the single epoch simulations), downsampled by a factor of 10 to a rate of 1 kHz prior to spectral analysis. The *set-point* of the trace was defined as the mean of the downsampled steady-state effector trace. The instantaneous phase $\phi_{whisk}(t)\;$was calculated relative to protraction onset using the Hilbert transform on the band-pass filtered, downsampled steady-state effector trace (same filter parameters as above), and ranged from 0 to π for protraction and from π to 2π for retraction.

As with experimental whisking data, power spectra for simulated effector segments (15 s duration) were computed for the unfiltered but mean-subtracted continuous traces using the multi-taper method with discrete prolate spheroidal sequences. Spectra were calculated using a time-half-bandwidth product of 15 and K=2NW−1=29 tapers to strictly control spectral leakage given the noisy inputs, for a frequency resolution of 1 Hz. The *dominant frequency* was defined as the frequency corresponding to the maximum power in the spectrum (Figs 2a, 4a, 4d, 4g, 4j). The dominant frequency for a simulation with no PB input is termed *intrinsic frequency*.

To quantify the synchronization between neuronal spiking and motor output in the model network, spectral coherence was calculated between the discrete spike times of the ${\text{vIRt}}^{\text{ret}}\;$and ${\text{vIRt}}^{\text{pro}}\;$populations and the continuous simulated effector position. Coherence was computed using the multi-taper method for point to continuous processes (Chronux toolbox function coherencycpt), using a time-half-bandwidth product of 15 and K=2NW−1=29 tapers as above. The magnitude of coherence (|C|) and the phase of coherence ($\phi_{C}$) were extracted at the *dominant frequency* of the effector spectrum. We computed both the individual coherence for each neuron and the aggregate coherence for the population by pooling all spike times across the population (Fig 4b, 4e, 4h, 4k). A neuron was considered significantly coherent if its coherence magnitude exceeded the 95% confidence interval estimated via the Jackknife method.

For “driven” simulations, to calculate the phase relationship between PB input current pulses and the effector signal, spectral coherence was calculated between the discrete PB input current pulse onset times and the continuous effector position, (Chronux toolbox function coherencycpt), using a time-half-bandwidth product of 15 and K=2NW−1=29 tapers, as above. Coherence phase was defined as effector protraction onset relative to PB input current pulse onset, so that negative values indicate an effector protraction phase lead. Coherence phase values at the PB pulse frequency, ($\varphi_{W-PB}$), were calculated by linear interpolation of the coherence phase vs. frequency curves.

#### Phase tuning curve.

To characterize the modulation of neuronal firing rates by the whisking cycle, *phase tuning curves* were calculated for the ${\text{vIRt}}^{\text{ret}}\;$and ${\text{vIRt}}^{\text{pro}}\;$populations. The instantaneous phase of the steady-state effector signal $\phi_{whisk}(t)$, was discretized into 16 equidistant bins. To account for potential non-uniformities in the phase distribution of the effector motion, we calculated the *occupancy time* for each bin, defined as the total duration the effector signal spent within that phase interval. The mean firing rate for each phase bin was then computed by summing the total number of spikes across all neurons in the population occurring within that bin, dividing by the total number of neurons (N), and normalizing by the occupancy time. This yielded a population-averaged firing rate (Hz) as a function of phase (Fig 4b, 4e, 4h, 4k) [66].

#### Spike Train Statistics.

Inter-spike intervals (ISIs) were calculated for all neurons in the ${\text{vIRt}}^{\text{ret}}\;$and ${\text{vIRt}}^{\text{pro}}$populations. To assess the variability of spiking regularity within bursts, we calculated the local coefficient of variation ($CV_{\text{2}}$). The $CV_{\text{2}}$ compares adjacent ISIs and is defined as:


$$\begin{array}{c} \begin{array}{c} CV_{\text{2}}=\frac{\text{2}\left|ISI_{i+\text{1}}-ISI_{i}\right|}{\left(ISI_{i+\text{1}}+ISI_{i}\right)} \end{array} \end{array}$$ (12)

To restrict this metric to intra-burst activity, ISIs exceeding half the dominant period were excluded from the calculation [24].

#### Intrinsic, driven, and root mean square whisk amplitudes.

For “Driven” simulations, the *driven whisk amplitude* was quantified using the mean peak amplitude of the first whisk following each PB current pulse onset time in the second half of the simulation. For simulations of “intrinsic” vIRt oscillations (no PB drive), the amplitude of the PB input current pulse train was set to 0 pA and the inter-pulse period was set to 200 ms. The *intrinsic amplitude* was quantified using the mean peak amplitude of the first whisk following each (zero-amplitude) PB current pulse onset time in the second half of the simulation. This definition ensures that oscillation amplitudes in “driven” and “intrinsic” simulations are calculated symmetrically. For Monte Carlo simulations (Fig 5c), confidence intervals for the mean *intrinsic amplitudes* and *driven whisk amplitudes* across the 30 repetitions were calculated using a bootstrap resampling method with 5,000 iterations, reporting the 2.5th and 97.5th percentiles.

In S1 Fig, because some combinations of intra-vIRt synaptic connectivity parameter sets resulted in effector output signals with low periodicity, the definition of *intrinsic amplitude* could not be applied consistently to this analysis. Therefore, for calculations in S1 Fig we define the *root mean square (RMS) amplitude* as the RMS value of the mean-subtracted effector output signal, and the *spectral peak signal-to-noise ratio (SNR)* as the ratio of the maximum observed signal spectral power at any sampled frequency between 0 and 25 Hz to the average spectral power over the frequency band from 0 to 25 Hz.

In Fig 7, for each intrinsic vIRt oscillation frequency $f_{intrinsic}$, the *amplification factor AF*, as a function of the PB pulse frequency is defined as:


$$\begin{array}{c} \begin{array}{c} AF\left(f_{\text{PB}}\right)=\frac{A\left(f_{\text{PB}}\right)}{A_{\text{intrinsic}}} \end{array} \end{array}$$ (13)

where $f_{\text{PB}}$ represents the PB pulse frequency, $A\left(f_{\text{PB}}\right)$ represents the *driven whisk amplitude*, and $A_{\text{intrinsic}}$ represents the intrinsic amplitude when there is no PB input.

#### Basal breathing cycle detection.

“Basal breathing” was defined by a breathing frequency of less than 3 Hz.

- *Experimental Data:* Intervals between detected respiratory peaks were used. For each respiratory peak, an associated inhalation-driven whisk was defined as the whisk peak occurring closest in time. A “basal breathing cycle” was included in the analysis if the inter-peak interval to the next respiratory peak exceeded 333 ms, the cycle contained at least 3 whisk peaks starting from the inhalation-driven whisk, and all peaks had valid amplitudes.
- *Simulated Data:* PB input current pulse cycle durations were used. The whisk peak immediately following the pulse was defined as the inhalation-driven whisk. A “basal breathing cycle” was included in the analysis if its duration exceeded 333 ms and contained at least 3 whisk peaks with all peaks having valid amplitudes.

#### Sniff onset window detection.

For sniffing onset window detection, “sniffing” was defined by a breathing frequency of greater than 4 Hz (a more sensitive threshold than the one used for spectral analysis).

- *Experimental Data:* Using the breathing signal, “sniff onset transition times” were first defined by the preceding valley time of each breathing peak where the preceding inter-peak interval is greater than 250 ms and the succeeding inter-peak interval is less than 250 ms. For each sniff onset transition, a “sniff onset window” was included in the analysis if there were at least 5 succeeding whisk peaks with all peaks having valid amplitudes.
- *Simulated Data:* A simulation had a sniff onset transition if a PB input current pulse cycle transitions from greater than 250 ms to less than 250 ms in a dual-epoch protocol. A “sniff onset window” was included in the analysis if there were at least 5 succeeding whisk peaks with all peaks having valid amplitudes.

#### Successive whisk amplitude metrics and statistics.

For correlating successive whisk amplitudes ($A_{n}$ vs.$A_{n+\text{1}}$), Pearson correlation coefficients ($r_{n+\text{1},n}$) were computed. To assess the statistical significance of the correlation, the correlation coefficient was transformed into a *t*-statistic using the following equation:


$$\begin{array}{c} t_{n+\text{1},n}=r_{n+\text{1},n}\sqrt{\frac{N-\text{2}}{\text{1}-{r_{n+\text{1},n}}^{\text{2}}}} \end{array}$$ (14)

where N represents the number of data points. The corresponding p-value was calculated based on the Student’s *t*-distribution with N− 2 degrees of freedom. For aggrega*t*ed statistics, the Fisher Z-transformation (inverse hyperbolic tangent, $z_{n+\text{1},n}=\text{atanh}\left(r_{n+\text{1},n}\right)$) was applied to approximate normality. For comparing successive whisk amplitudes $A_{n+\text{1}}/A_{n}$, the logarithmic decrement calculated as $d_{n+\text{1},n}=\;\text{ln}\left(A_{n+\text{1}}/A_{n}\right)$ was used to approximate normality.

For experimental data, as the epoch start and end times were arbitrary, logarithmic ratios were computed for each detected analysis window and pooled for statistical comparison. Using a geometric mean of the p values from three different normality tests (the Lilliefors test, the Anderson-Darling test and the Jarque-Bera test), a weighted majority of the pooled logarithmic decrements $d_{n+\text{1},n}$ (with higher weight given to earlier decrements) reached a significance level of p < 0.05 (failed the normality test), so a signed-rank test was used to compare the median $\tilde{d_{n+\text{1},n}}\;$against zero.

For simulated (Monte Carlo) data, logarithmic ratios $d_{n+\text{1},n}$ were computed for each detected analysis window. Then, the arithmetic mean was computed over each simulation (with different randomized seeds) as $\overset{―}{d_{n+\text{1},n}}\;$before pooling for statistics. Fisher Z-scores of amplitude correlations $z_{n+\text{1},n}$ were computed for each simulation and pooled for statistics. These pooled amplitude metrics all passed the normality test described above, so a Student’s *t*-test was used to compare the means $\overset{―}{z_{n+\text{1},n}}$ and $\overset{―}{\overset{―}{d_{n+\text{1},n}}}$ against zero and compute 95% confidence intervals. All probability values reported are two-tailed, wi*t*h statistical significance defined as p < 0.05. Aggregated *average amplitude ratios* $\overset{―}{\overset{―}{A_{n+\text{1}}/A_{n}}}$ and *average correlation coefficients* $\overset{―}{r_{n+\text{1},n}}$ were computed by $\overset{―}{\overset{―}{A_{n+\text{1}}/A_{n}}}=exp(\overset{―}{\overset{―}{d_{n+\text{1},n}}})\;$and $\overset{―}{r_{n+\text{1},n}}=tanh(\overset{―}{z_{n+\text{1},n}})$, respectively.

#### Whisking and sniffing amplitude and phase relationships.

For experimental data, we calculated the Pearson correlation coefficient between *mean sniff frequency* and *mean whisk amplitude* per sniffing segment, and we evaluated significance with a t-test (MATLAB function corr). We calculated the circular-linear correlation coefficient between whisking-sniffing coherence phase at the mean sniff frequency ($\varphi_{WS}$) and the mean sniff frequency, and between $\varphi_{WS}$ and mean whisk amplitude (see Section **Spectral analysis for experimental data**). We calculated the circular mean and circular standard deviation for the set of $\varphi_{WS}$ values from each sniffing segment. Circular statistics were calculated using the MATLAB CircStat toolbox [32].

### Estimation of PB spiking to inhalation phase relationship

We estimated the approximate phase delay between PB burst onset and inhalation onset during sniffing, as measured via airflow-induced temperature change, from published data [14]. There is substantial variability in the preferred phase of burst activity (burst midpoint) in inhalation-locked units in the approximate region of PB that ranges from phase leads of π/2–0 [14]. Assuming burst duty cycles between 25% and 50%, this would add an additional phase lead of π/4 to π/2 from burst onset to burst midpoint, yielding a total range of π to π/4. Since both the preferred phase and duty cycle may be cell-type-specific, and the cell type of vIRt-projecting PB neurons is uncharacterized, we consider this entire range. We assume a constant phase delay for this estimation, and note that a constant time delay would instead result in a sniff frequency-dependent phase shift, but would be unlikely to result in a change in the phase-amplitude relationship.

#### Principal component analysis.

For the undriven vIRt/FMN network model (Fig 6a), it was assessed whether the values of the input current parameters μ, σ, and F (see **External Inputs and Simulation Protocol**) that resulted in simulated *intrinsic amplitudes* in the “target range” co-varied (see **Network Entrainment and Detuning Protocol**). For this assessment, a matrix $X_{inputs}$ of size $N_{triplets}$ x 3 was constructed, with each row corresponding to a μ, σ, F triplet that results in a simulated oscillation in the target range. The data were then normalized by computing the z-scores of the values in each of the columns of $X_{inputs}$ separately. The principal components (PCs) of the resulting matrix were computed using the MATLAB pca() function. The fraction of the variance explained by the *i-*th PC, $f_{{explained}_{i}}$, was calculated as:


$$\begin{array}{c} \begin{array}{c} f_{{explained}_{i}}=\frac{v_{i}}{\sum_{i}v_{i}}\; \end{array} \end{array}$$ (15)

where $v_{i}$ represents the variance of the *i-*th PC. The Pearson correlation coefficients between PC1 and each of the variables μ, σ, F (Fig 6d), as well as the corresponding *intrinsic frequency* estimates (Fig 6e) were calculated using the MATLAB corr() function.

Statistical significance of the values of $f_{{explained}_{i}}$ were estimated by performing a permutation test with 10,000 iterations and estimating the p-value as the fraction of values in the simulated null distribution that exceeded the actual $f_{{explained}_{i}}$. A permutation test was also used to assess statistical significance of the correlation coefficients between the parameters μ, σ, F, and PC1 (Fig 6d), and between the estimated *intrinsic frequency* and PC1 (Fig 6e). 95% confidence intervals of $f_{{explained}_{i}}$ and the correlation coefficients between PC1 and the parameters μ, σ, F, were calculated by bootstrapping with 10,000 iterations, reporting the 2.5th and 97.5th percentiles.

#### Phase response to basal breathing.

The phase response of the whisking cycle to simulated basal breathing pulses was calculated as follows: the whisk cycle phase was defined based on the valley (protraction onset) times. The breathing perturbation time $t_{breath}$ was defined as the PB input current pulse start time. The whisk phase shift $\Delta \phi_{whisk}$ in response to the breathing perturbation $\phi_{breath}$ was defined as the proportional change in the cycle duration relative to the unperturbed period:


$$\begin{array}{c} \phi_{breath}=\text{2}\pi \cdot \text{mod}\left(\frac{t_{breath}-t_{whiskbefore}}{T_{\text{0}}},\;\text{1}\right) \end{array}$$

$$\begin{array}{c} \begin{array}{c} \Delta \phi_{whisk}=\text{2}\pi \left(\frac{T_{\text{1}}-T_{\text{0}}}{T_{\text{0}}}\right) \end{array} \end{array}$$ (16)

where mod() is the modulo function, $T_{\text{0}}=t_{whiskbefore}-t_{\text{2}whisksbefore}$ is the unperturbed inter-valley interval preceding the respiratory perturbation and $T_{\text{1}}=t_{whiskafter}-t_{whiskbefore}$ is the perturbed inter-valley interval straddling the respiratory perturbation. Positive values of $\Delta \phi_{whisk}$ indicate a prolongation of the cycle (phase delay), while negative values indicate a shortening of the cycle (phase advance).

### Graphical User Interface (GUI)

To facilitate rapid parameter exploration and real-time visualization of network dynamics, a custom Graphical User Interface (GUI) was developed using MATLAB App Designer. The GUI provides interactive fields for modifying all simulation and analysis parameters as described above, allowing the user to run single simulations or sets of simulations (varying a single parameter such as the randomization seed, square wave input period, etc.) with direct visualization of output for immediate feedback. Sample outputs include:

- **Sample Traces:** Displays real-time voltage traces ($V_{m}$) for representative neurons from each population aligned with the external PB current inputs and continuous trace of the effector position (as in Fig 3c and 3e).
- **Network Activity:** Renders a spike raster plot sorted by population, synchronized with the continuous trace of the effector position (as in Figs 3d, 3f and 8a).
- **Analysis Plots:** Whisk log decrement jitter plots (as in Fig 8e and 8f), amplitude scatter plots (as in Fig 8b) and phase response curves (as in Fig 8d).

### Code

All simulations and analysis were performed in MATLAB R2023a, R2025a or b (MathWorks) using the Statistics and Machine Learning Toolbox, Signal Processing Toolbox, Image Processing Toolbox, Parallel Computing Toolbox, the Chronux toolbox version 2.12 (http://chronux.org, released 2018-10-25), the CircStat toolbox version 1.21.0.0 (https://www.mathworks.com/matlabcentral/fileexchange/10676-circular-statistics-toolbox-directional-statistics, released 2012-06-08), and custom scripts.

### Declaration of generative AI and AI-assisted technologies

During the preparation of this work the authors used Google Gemini to assist in generating prototype MATLAB code, with extensive author oversight, revision, validation, organization, and documentation of the implementation. After using this tool, the authors reviewed and edited the content as needed and take full responsibility for the content of the published article.

## Supporting information

S1 FigIntrinsic oscillation dynamics in the model whisking CPG network with no periodic drive depend on intra-vIRt synaptic currents.(a) Heatmaps of effector *intrinsic frequency* (top left), *RMS amplitude* (bottom left)*, set-point* (top right) and *spectral peak signal-to-noise ratio (SNR)* (bottom right) (see **Materials and Methods** for definitions) for a model vIRt/FMN network with no periodic drive. Horizontal axes represent the intra-pool synaptic current values ($W^{rr}$and $W^{pp}$), and vertical axes represent the inter-pool synaptic current values ($W^{rp}$and $W^{pr}$). For illustration of synaptic current values that resulted in whisking-like oscillations, white squares indicate simulations for which the oscillation *intrinsic frequency* and *spectral peak SNR* exceeded threshold values of 4 Hz and 5, respectively. * indicates simulations for which example traces are shown in S1b Fig. ^x^ indicates values of $W^{rp},$ $W^{pr},\;W^{rr},$ and $W^{pp}$ used in subsequent simulations throughout the manuscript. **(b)** Example traces of model effector output signals with the parameter sets indicated by * in **panel a.**(EPS)

S2 FigFrequency-selective amplification and whisking-to-PB phase in the model whisking CPG network with periodic drive varies with PB input strength.(a) Plot of the mean amplitude of the first whisk following each $I_{ext}^{PB}$ pulse at steady state (*driven whisk amplitude*) for simulations of the network in Fig 3a at $I_{ext}^{PB}$ pulse frequencies ranging from 1-10 Hz for varying values of $W^{rB}$ (in pA) as indicated in the legend. (see **Materials and Methods**). The horizontal and vertical black bars represent the mean intrinsic amplitude and the intrinsic frequency, respectively, of simulations in which $I_{ext}^{PB}$ = 0. **(b)** Plot of the phase of coherence between PB pulse onset times and the effector signal, calculated at the PB pulse frequency ($\varphi_{W-PB}$), vs. PB pulse frequency. Negative values of $\varphi_{W-PB}$ indicate that protraction onset leads PB pulse onset. The vertical black bar represents the intrinsic frequency, as in **panel a**. The horizontal black dotted line represents a phase shift of 0 radians. Colors represent different $W^{rB}$ values, as in **panel a.**(EPS)

S3 FigTime course of effector amplitude changes in successive cycles in the model whisking CPG network following low-frequency PB current pulses (a) Sample segment of a simulation of the network in Fig 3a with 30 seconds of $I_{ext}^{PB}\;$ as a pulse train with pulse duration 50 ms, amplitude 500 pA, and frequency 0.5 Hz (sub-basal breathing frequency).Effector position (blue) and PB current pulse stimulation times (gray) are shown. (b) Driven (first) whisk amplitude over all $I_{ext}^{PB}$ pulses in each of 30 Monte Carlo simulations (purple dots). The gray line indicates the mean of these means over all 30 simulations. **(c)** Distributions of log ratios $d_{n+\text{1},n}=\;\text{ln}\left(A_{n+\text{1}}/A_{n}\right)$ of successive whisk amplitudes, where A represents the amplitude and n represents the nth whisk after a stimulation at 0.5 Hz. Each gray dot represents the mean log ratio for all the $I_{ext}^{PB}$ pulses at steady state in one simulation. Red circles indicate the mean of the mean log ratios $\overset{―}{\overset{―}{d_{n+\text{1},n}}}$ over the 30 Monte Carlo simulations, and red bars indicate 95% confidence intervals of the mean. Average amplitude ratios $\overset{―}{\overset{―}{A_{n+\text{1}}/A_{n}}}=exp(\overset{―}{\overset{―}{d_{n+\text{1},n}}})\;$are displayed on the top. Statistical significance (Student’s *t*-test on $\overset{―}{\overset{―}{d_{n+\text{1},n}}}\text{≠}\text{0}$): *** p < 0.001, ** p < 0.01, * p < 0.05.(EPS)
